# Impact of Nitrogen Sparging on Chemical and Sensory Characteristics of *Verdejo* and *Sauvignon blanc* Wines

**DOI:** 10.3390/foods14132272

**Published:** 2025-06-26

**Authors:** del Barrio-Galán Rubén, del Alamo-Sanza Maria, Martínez-Gil Ana María, González-Lázaro Miriam, Nevares Ignacio

**Affiliations:** 1 Department of Analytical Chemistry, Universidad de Valladolid, 34001 Palencia, Spain; rube.barrio@uva.es (d.B.-G.R.); anamaria.martinez.gil@uva.es (M.-G.A.M.); 2 UVaMOX-Unidad Asociada al CSIC, Universidad de Valladolid, 34001 Palencia, Spain; 3 Instituto de Ciencias de la Vid y del Vino (ICVV), 26007 Logroño, Spain; miriam.gonzalez@icvv.es; 4 Department of Agricultural and Forestry Engineering, Universidad de Valladolid, 34001 Palencia, Spain

**Keywords:** sparging, nitrogen, oxygen, deoxygenation, volatile compounds, sensory profile

## Abstract

Sparging is a common technique in wineries that consists of injecting a gas, normally before bottling, in order to displace the dissolved oxygen in the wine and prevent oxidation. The objective of this study was to examine the effect of sparging on wines with three different levels of dissolved oxygen and the evolution of the chemical parameters in a bottle. This study was carried out on two white wines, *Verdejo* and *Sauvignon blanc*. The results indicated that sparging did not immediately affect the chemical parameters in the white wines, but it did affect their evolution in bottles, with a greater effect found in the *Sauvignon blanc* wines than in the *Verdejo* wines. Sparging, which was carried out to remove oxygen from the wines, had a protective effect on their color during the time in the bottles, preventing a more rapid decrease in free SO_2_. The effect of sparging on the volatile compounds of the wines was more evident in the *Sauvignon blanc* wines, which showed a reduction in their content, possibly due to carry-over when the N_2_ was applied. With regard to the effect of sparging on the sensory profile of the wines, no immediate effect was found. However, the wines with a DO content of 6 and 8.4 mg/L to which sparging was applied evolved better in the bottles than the deoxygenation wines, showing more fruity notes and fewer oxidized and phenolic aromas (mainly in the *Verdejo* wines).

## 1. Introduction

It is well known in the winemaking world that a high concentration of dissolved oxygen (DO) can lead to the oxidation of wines; this is especially critical in white wines [1]. Throughout the winemaking process, it is very likely that oxygen will be incorporated into the wine due to its contact with atmospheric air. The total amount of oxygen that is incorporated into the wine at the end of its production may exceed the amount that does not affect, or that minimally affects, the chemical and sensorial characteristics of the wine [2,3]. Therefore, it is usually recommended to eliminate this excess oxygen before bottling to avoid future problems during the wine’s storage in bottles. The most common way to eliminate excess DO is by continuously injecting nitrogen (N_2_), a technique known as “sparging”. The use of this technique in winemaking dates back to 1960 [4]. In addition to N_2_, other inerting gases can be used, such as argon (Ar), carbon dioxide (CO_2_) [5], or a mixture of N_2_ and CO_2_. Saa et al. (2013) [5] studied the effect of CO_2_ bubbling on the oxygen content by simulating the hydrodynamic conditions of wine fermentation, and determined that this bubbling allowed the concentration of dissolved oxygen to be reduced to equilibrium, mainly due to the dilution effect. However, a previous study showed that, from a cost–benefit point of view, N_2_ is the best inert gas for reducing the oxygen content in finished wines that are to be bottled [2]. In addition, from a sustainability perspective, the use of nitrogen (N_2_) for sparging can be considered an efficient and environmentally responsible practice, particularly in wineries equipped with on-site nitrogen generators. These systems typically produce nitrogen for the continuous generation of high-purity N_2_ from ambient air without the need for transportation or storage of gas cylinders. This not only reduces the carbon footprint associated with logistics but also minimizes waste and improves operational safety. Moreover, the energy consumption of modern generators is relatively low, making them a viable option for wineries aiming to implement sustainable and cost-effective oxygen management strategies during winemaking and bottling.

The sparging technique is based on Henry’s law [1,6], and it is observed that when N_2_ is introduced into wine in large quantities, the other dissolved gases, such as oxygen (O_2_) and CO_2_, tend to exchange with the N_2_ present in the bubbles, which leads to their elimination from the wine by displacement with the N_2_ when they exceeds their solubility in the liquid [6]. This method has proven effective in eliminating these gases from wine, as well as in reducing excess SO_2_ and certain sulfurous aromas that come from reductive processes [5,6]. However, there is still no conclusive information on how this practice can influence the composition of wine, nor on the physical and operational factors that can affect the effectiveness of bubbling N_2_ [7]. Some authors have concluded that the effect of degassing with N_2_ or other inert gases can vary depending on the type of wine [2]. However, there are studies on micro-oxygenation that have identified certain factors that can affect the solubility of oxygen in wine. A high sugar content decreases the rate of oxygen transfer, while a low ethanol content considerably favors the transfer of oxygen to the wine [8,9]. These studies agree that in wines with a high content of inert gases, such as CO_2_, it favors the desorption of oxygen from the wine matrix, decreasing the oxygen transfer rate [8,9].

On the other hand, the potential effects of sparging on the volatile compounds responsible for the aroma of wines are still relatively unknown, and there is speculation about how this technique can influence these compounds that are important for the quality of wine. Some authors indicate that sparging, unless carried out with caution, can displace certain compounds that have a positive impact on the flavor and aroma of wine such as majority volatiles (2-phenylethanol) and minority volatiles (ethyl esters terpenes and vanillic derivatives), but these effects depend on the wine matrix and the gas used [2]. However, a recent study by Walls et al. (2022) [7] on white wines found that there were no significant modifications to the volatile compounds analyzed, such as varietal thiols, esters, volatile fatty acids and alcohols, after removing dissolved oxygen from the wine under certain conditions (using N_2_ and a mixture of N_2_ and CO_2_ (70:30) for sparging; removing an oxygen content of 3 mg/L from white wines; and using two different flows: 120 and 240 mL/L wine/minute). However, del Barrio-Galán et al. (2024) [2] showed that the use of N_2_, CO_2_ and Ar to eliminate a DO concentration of 3 mg/L in white and red wines produced, in some cases, a loss of volatile compounds that contribute to the sensory profile of wines, such as ethyl esters, acetate esters, terpenes, vanillin derivatives and, in other cases, an increase in alcohols, aldehydes and volatile phenols.

For greater efficiency during sparging, it is recommended to perform it using the lowest possible gas/liquid ratio. In addition, factors such as the size of the gas bubbles, the ratio between the flow rate of the inert gas and the flow rate of the wine (especially if it is carried out during racking), the application time (which refers to the duration of contact between the inert gas and the wine), the temperature of the wine, the pressure at which the gas is applied, the frequency of application, the initial amount of oxygen dissolved in the wine, the design of the winemaking facility and the equipment used are key aspects to consider [10,11,12,13,14,15]. According to Girardon [11], the efficiency of the process of eliminating dissolved oxygen in wine can be improved by introducing spiral turbulence with an in-line vortex, which could increase efficiency by up to 90%. Other researchers have used a mixer–agitator to promote the diffusion of inert gas in white wine, thus promoting the elimination of 3 mg/L of dissolved oxygen [7]. However, it is crucial to avoid excessive agitation, as this can generate a heterogeneous bubble pattern instead of the homogeneous pattern that is produced with lower gas flow rates and is characterized by smaller bubbles that rise evenly from the diffuser [16].

The effectiveness of sparging also depends on the wine matrix. Thus, the most appropriate time to perform sparging is at the end of the winemaking process, when the wine has a lower protein content, which is relevant for the desorption of oxygen when applying N_2_ [17]. In addition, factors such as the levels of ethanol, glycerol, sugar and dry extract can influence the viscosity of the wine, thus affecting the oxygen desorption process. In aqueous solutions such as wine, the presence of phenols, acids, alcohols, surfactants and ions also impacts this process [17].

The two most common methods for removing dissolved oxygen from wine are to bubble the inerting gas into a tank of wine, forming a column of inerting gas bubbles and/or to bubble the gas in the line as the wine is being moved [3,4,15,17,18]. Other methods are used to eliminate oxygen dissolved in the water, such as boiling at atmospheric pressure and boiling at reduced pressure [19]. However, these processes require a greater energy expenditure compared to sparging and can be detrimental to the quality of the wine, as they can destroy positive aromatic components and cause the loss of ethanol [20].

Recently, the wine sector has started to use membrane contactors [21,22,23,24], which are considered less invasive because they do not eliminate aromatic compounds from the wine. However, their use involves a high cost and requires the wine be well filtered before coming into contact with the membrane and regular cleaning of the membrane [2,3,4,5,6,7,8,9,10,11,12,13,14,15,16,17,18,19,20,21,22,23,24].

There are few works in the literature and in many of them, only the removal of a certain concentration of oxygen was studied. For this reason, and based on all the above, this study analyzed the effect of removing oxygen by sparging with N_2_ before bottling on the physicochemical properties and sensory profile of white wines with different oxygen contents.

## 2. Materials and Methods

### 2.1. White Wines and Sparging Treatment

Wines of the *Verdejo* and *Sauvignon blanc* varieties, vintage 2023 from the region of Castilla y León (Spain), were used. The treatments for the removal of DO in the wine were simultaneously carried out in duplicate using a volume of 3 L of wine placed in two identical Plexiglas tubes with a capacity of 4 L and a diameter of 5 cm. The tests were carried out at a temperature of 15.5 °C. The oxygen content was measured with a DP-PSt6 immersion probe connected to a measuring device (PreSens GmbH, Regensburg, Germany), which was submerged 10 cm below the surface of the wine. All the probes were periodically calibrated according to the manufacturer’s instructions.

The methodology consisted of simulating different levels of dissolved oxygen in white wines by means of air uptake until they reached a certain partial pressure of O_2_ (pO_2_) (60 hPa (wine W3), 120 hPa (wine W6) and 180 hPa (wine W8.4); if we consider the solubility of O_2_ in water, these would be equivalent to 3, 6 and 8.4 mg/L, respectively) and then reduced this level of dissolved oxygen following a previously tested methodology with certain modifications [2]. The removal of dissolved oxygen was carried out by bubbling N_2_ at a flow rate of 0.03 L/minute until a pO_2_ ≤ 6 hPa was reached (which is equivalent to an oxygen content of less than 0.3 mg/L if we consider the solubility of oxygen in water) to obtain the deoxygenated wines DEOX 3, DEOX 6 and DEOX 8.4. The N_2_ used came from a N_2_ generator (PSA Technology, model Nitrogen 15; Sysadvance, Póvoa de Varzim, Portugal) that is installed in the experimental winery of the Higher Technical School of Agricultural Engineering in Palencia, University of Valladolid. To reach the desired levels of dissolved oxygen (DO), the uptake of atmospheric air and the removal of nitrogen were carried out using a porous metal diffuser with a pore size of 2 microns and a contact surface area on the wine of 11.9 cm^2^, as these characteristics allowed for a more homogeneous bubble size. Therefore, the contact surface area per liter of wine was 4.75 cm^2^/L.

Once the sparging of the wines at the three dissolved oxygen levels was finished, they were bottled in 0.375 L bottles with synthetic stoppers, which were previously inertized with N_2_. Synthetic stoppers were selected to ensure consistency in the oxygen transmission rate (OTR) across all samples, minimizing variability that could arise from the natural heterogeneity of cork. This choice also allowed us to better isolate the effects of dissolved oxygen and the sparging treatments. The wines were kept in controlled humidity (between 72.5 and 76.5%) and temperature (between 15.5 and 16.5 °C) conditions during their storage in bottles for 1, 3 and 6 months.

### 2.2. Chemical Analysis of White Wines

The classic oenological parameters (alcohol content, pH, total acidity, volatile acidity, free SO_2_ and total SO_2_ contents, TPI and color) were analyzed according to the official methods of the OIV (2022) [25]. Tartaric esters and flavonols were determined according to the methods of Mazza et al. [26]. The CieLAB color coordinates, L*, a* and b*, were measured following the MSCV methodology [27]. In addition, the absorbance spectrum was measured between 330 nm and 700 nm for all wines. All spectrophotometric analyses were performed with a Perkin Elmer LAMBDA 25 UV/vis spectrophotometer (Waltham, MA, USA).

The minority volatile compounds were analyzed by GC-MS (Agilent, Palo Alto, CA, USA) according to the methodology established in Garde-Cerdán et al. [28]. Briefly, the extraction of wine volatile compounds was performed by stirring 8 mL of each sample (for 15 min) with 400 μL of dichloromethane (Merck, Darmstadt, Germany). After cooling for 10 min at 0 °C, the organic phase was separated by centrifugation (5031× *g*, 10 min, 4 °C), and the extract was recovered and transferred into a vial. The following compounds were identified and quantified: alcohols (isobutanol, 1-butanol, isoamyl alcohols, 3-methyl-1-pentanol, benzyl alcohol, phenylethyl alcohol, 1-hexanol, cis-3-hexen-1-ol, metionol and tyrosol), ethyl esters (ethyl hexanoate, ethyl octanoate, ethyl decanoate, monoethyl succinate, diethyl succinate and ethyl lactate), acetate esters (isoamyl acetate, hexyl acetate and phenylethyl acetate), fatty acids (propanoic acid, butyric acid, isobutyric acid, isovaleric acid hexanoic acid, octanoic acid and decanoic acid), an aldehyde (benzaldehyde), vanillic derivatives (methyl vanillate, vanillyl acetone and acetovanillone), a terpene (trans-geraniol), a lactone (butyrolactonone), a norisoprenoid (β-damascenone) and phenols (eugenol and 4-vinylguaiacol). 2-Octanol (Sigma-Aldrich, Madrid, Spain; concentration: 2.5 g/L in ethanol) was used as an internal standard. The identification of volatile compounds was carried out using the NIST library and through comparisons with the mass spectra of available standards (Sigma-Aldrich, Madrid, Spain). A semi-quantification was carried out, relating the areas of each volatile compound with the area from a known concentration of the internal standard.

All the analyses were carried out in duplicate.

### 2.3. Sensory Analysis of White Wines

The sensory analysis was carried out in the tasting room of the Higher Technical School of Agricultural Engineering of Palencia, University of Valladolid, which complies with the UNE-EN ISO 8589:2010/A1:2014 regulations [29]. This analysis was conducted with a panel of expert tasters from the region of Castile and León who are accustomed to tasting this variety of wine. The panel was made up of 11 people, 6 men and 5 women, who are oenologists and researchers from wineries in Castile and León, aged between 26 and 67. The bottles were opened 20 min before the tasting. The samples were kept at a constant temperature (14–15 °C) to facilitate their tasting. The glasses were identified with randomly chosen three-digit codes. The glasses were filled with about 30 mL of wine and were randomly presented to the tasters so that each taster had the wines placed in a different order. The following aspects were evaluated in the visual phase: color intensity, and yellow, green and golden tones; in the olfactory phase, they were fruity, floral and herbaceous/green/vegetal aromas, oxidized and phenolic smells and olfactory intensity; and in the gustatory phase, they were alcohol, acidity, bitterness, persistence and balance. These aspects were scored using a structured 5-point scale. In each session, the wines with different levels of dissolved oxygen (W 3, W 6, W 8.4) and the deoxygenated wines (DEOX 3, DEOX 6, DEOX 8.4) were tasted.

The wines were analyzed and tasted at four different times: initially (freshly bottled) and after 1, 3 and 6 months in a bottle.

### 2.4. Statistical Analysis

All the data were analyzed using analysis of variance (ANOVA) and the least significant difference (LSD) test at a significance level of *p* < 0.05. The statistical analysis was carried out using the statistical program “InfoSat version 2012p” (FCA-National University of Córdoba, Córdoba, Argentina). Multivariate analysis of variance (MANOVA) was performed with the program Statgraphics Centurion 19 (Statgraphics Technologies Inc., The Plains, VA, USA). A principal component analysis (PCA) was performed with the variables that changed the most modified after sparging.

## 3. Results and Discussion

### 3.1. Efficacy of the Use of N_2_ for DO Removal in White Wines

**Figure 1:**
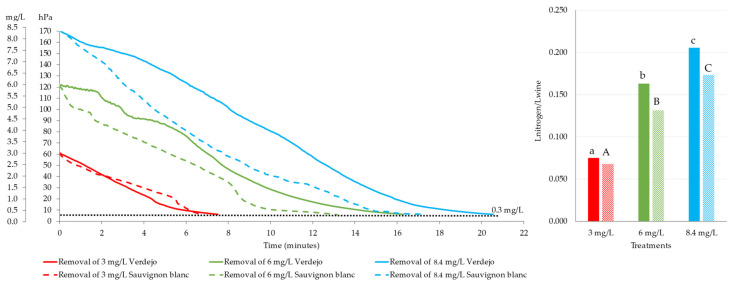
Deoxygenation kinetics for *Verdejo* and *Sauvignon blanc* wines at different oxygen concentrations. Different lowercase letters for *Verdejo* and uppercase letters for *Sauvignon blanc* indicate statistically significant differences.

Figure 1 shows that the more dissolved oxygen the wine has, the longer the N_2_ had to be applied, and therefore the greater the quantity needed to displace the DO. Thus, the quantity of N_2_ used to remove 3 mg/L of DO from wine was 0.075 L of N_2_/L of wine for the *Verdejo* and 0.068 L of N_2_/L of wine for the *Sauvignon blanc*. The volume of N_2_ to remove 3 mg/L of DO was very similar to that of another previous study, where different inerting gases were used to evaluate their effectiveness in removing 3 mg/L of DO from white and red wines [2]. To remove higher amounts of DO (6 and 8.4 mg/L), it was necessary to use more N_2_ for the *Verdejo* wines (0.163 L and 0.206 L N_2_/L wine) than for the *Sauvignon blanc* wines (0.132 L and 0.173 L N_2_/L). Depending on the amount of oxygen to be removed, it was necessary to use between 10 and 25% more N_2_ for the *Verdejo* wine than for the *Sauvignon blanc*. As discussed in Sutton et al. [14], the effectiveness of sparging will depend on the wine matrix, since an increase in the levels of ethanol, glycerol, sugar, dry extract, phenols, acids, alcohols, etc., can affect this process due to an increase in viscosity. However, in this study, for the *Sauvignon blanc* wines, which have higher levels of alcohol, phenolic compounds, etc., than the *Verdejo* wines, a smaller amount of N_2_ was needed. These differences were not due to the alcohol content but could be due to other factors such as residual sugar, glycerol, or dry extract.

### 3.2. Effect of Sparging on the Chemical Parameters of White Wines with Different DO Levels

The results of the analyses carried out on the *Verdejo* and *Sauvignon blanc* wines are presented in Table S1. Table 1 shows the results of the multifactorial ANOVA (MANOVA) taking into account the time spent in the bottles (time factor: 0, 1, 3 and 6 months), if sparging was carried out before bottling (sparging factor: yes or no) and the level of dissolved oxygen in the wines (O_2_ level: 3, 6 and 8.4 mg/L). Time was the factor that affected the parameters the most for both the *Verdejo* and *Sauvignon blanc* wines. The second most influential factor depended on the variety: the O_2_ level (11 parameters) for the *Verdejo* and whether sparging was performed or not (13 parameters) for the *Sauvignon blanc*.

#### 3.2.1. Effect of Sparging on Basic Chemical Parameters

**Figure 2:**
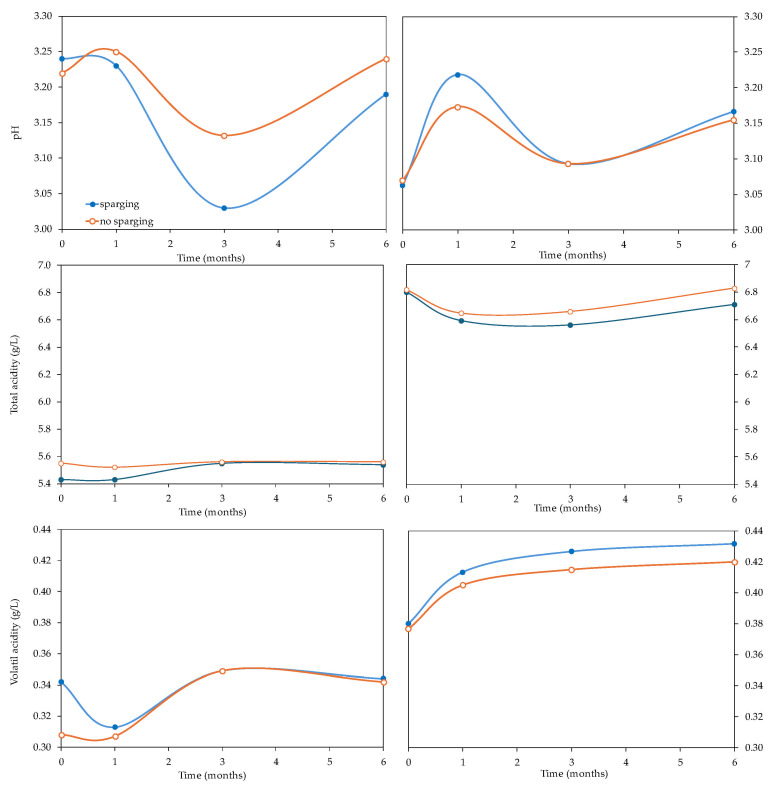
Evolution of pH, total and volatile acidity of *Verdejo* and *Sauvignon blanc* wines with and without sparging treatment over 6 months in bottles.

**Figure 3:**
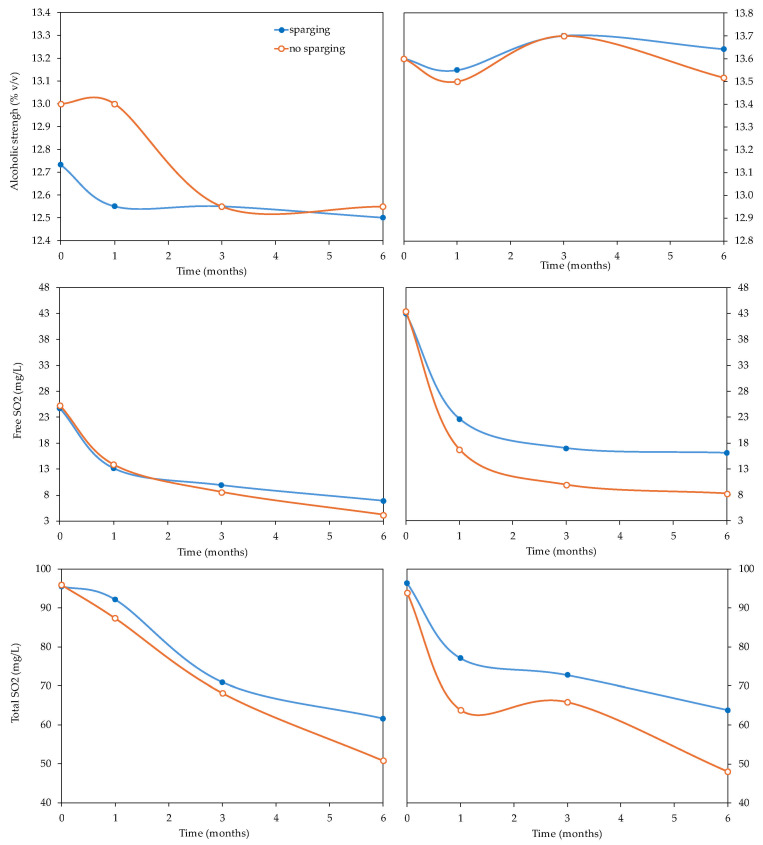
Evolution of the alcohol, free SO_2_ and total SO_2_ contents of *Verdejo* and *Sauvignon blanc* wines with and without sparging treatment over 6 months in bottles.

Bottle aging and sparging with N_2_ were the factors that affected the classic parameters the most in both varieties (Table 1). Figure 2 and Figure 3 show the evolution of the classic parameters of the *Verdejo* and *Sauvignon blanc* wines with and without sparging treatment over 6 months.

The pH of the freshly deoxygenated *Verdejo* wines ranged from 3.22 to 3.25 (0 M). In general, it was seen that the DEOX 3, DEOX 6 and DEOX 8.4 wines had pH levels 0.02 units higher than the corresponding W3, W6 and W8.4 wines at the time of bottling. However, the relationship was reversed with the passage of time in bottles (Figure 2), with time and sparging having an effect on this parameter (Table 1). After 3 months, the differences between the wines with and without sparging were 0.11–0.13 units, although after 6 months in bottles, the values were very similar between those that had or had not undergone sparging (Table S1). In the first months after bottling, certain differences could be seen due to the sparging treatment, but these disappeared over time. The same happened in the *Sauvignon blanc* wines, but the differences were seen in the first weeks and, in this case, the wines from which the DO was removed were the ones that showed higher pH values (Figure 2). However, the total acidity was somewhat higher in the wines of both varieties when sparging had not been carried out (Figure 2 and Table S1), but these differences, which were less than 0.14 g/L in both wines, indicate that sparging had little oenological effect on total acidity.

The volatile acidity of the *Verdejo* wines ranged between 0.31 and 0.35 g/L (Table S1) after sparging, with significant differences found between the wines with and without sparging, as well as differences due to the oxygen level of the wines and the time spent in the bottles (Table 1). The largest differences were found immediately after sparging, with the wines without sparging showing lower values than the wines with sparging. In the *Sauvignon blanc* wines, the volatile acidity values after sparging ranged between 0.37 and 0.40 g/L (Table S1), showing significant differences with bottle age (Table 1). Therefore, the values obtained after sparging did not imply an oenological problem since these values were within the normal range for wines.

Sparging can lead to a carry-over of volatile substances. The alcohol content showed little change, ranging from 12.50% to 12.80% *v*/*v* in the different *Verdejo* wines and at the different times of analysis, with no significant differences in the period studied. In *Sauvignon blanc* wines, this parameter varied between 13.5 and 13.7% *v*/*v,* so sparging did not affect the alcohol content of the wines, with differences of less than 0.5% *v*/*v* (Figure 3).

Sparging also did not have an immediate effect on the sulfur dioxide content of the wines, with levels of free F-SO_2_ between 24 and 28 mg/L in the *Verdejo* wine and 42 to 44 mg/L in the *Sauvignon blanc* (Figure 3). As expected, the evolution of the wines in bottles caused a decrease in the F-SO_2_ content, with differences between the wines bottled after sparging and those bottled without. In addition, as can be seen in Figure 3, as time in the bottles increased, the differences in F-SO_2_ content between the two wines increased. In general, the wines bottled without oxygen removal had a lower F-SO_2_ content. In contrast, the wines bottled after sparging (DEOX 3, DEOX 6 and DEOX 8.4) maintained higher F-SO_2_ values, with the difference increasing with the time spent in the bottles. Boulton et al. (1996) [29] indicate that, theoretically, 1 mg/L of oxygen can consume 4 mg/L of SO_2_, so this immediate loss of free SO_2_ due to DO at bottling should also be taken into account when levels of SO_2_ at bottling are determined; thus, the *Verdejo* wines would lose between 16 and 23 mg/L of F-SO_2_ and the *Sauvignon blanc* wines would lose between 24 and 37 mg/L of F-SO_2_. In general, the T-SO_2_ levels also decreased more clearly in the wines that were bottled with higher levels of oxygen compared to those that were deoxygenated.

These results obtained for the *Verdejo* and *Sauvignon blanc* wines indicate that the process of sparging before bottling does not substantially modify the general characteristics of the wines. It is important to emphasize that applying sparging just before bottling allows the F-SO_2_ level to be maintained during the time the wine spends in bottles, thus better protecting these wines against oxidation.

#### 3.2.2. Effect of Sparging on Phenolic Compounds in Wines

**Figure 4:**
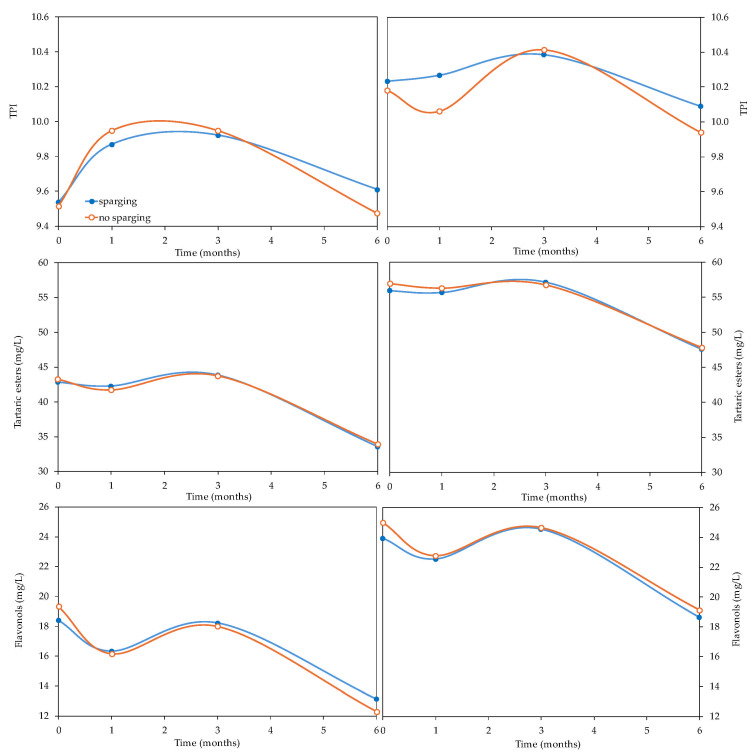
Evolution of the phenolic families of *Verdejo* and *Sauvignon blanc* wines with and without sparging treatment over 6 months in bottles.

Figure 4 shows the evolution of phenolic compounds in the *Verdejo* and *Sauvignon blanc* wines, with or without sparging before bottling, over 6 months. Initially, the TPI, and tartaric ester and flavonol contents were clearly higher in the *Sauvignon blanc* wines than in the *Verdejo* wines. The contents of these compounds in the white wines of the region of Castile and León is usually higher in *Verdejo* wines than in *Sauvignon blanc* wines [30]. This discrepancy may be attributed to several factors such as the vintage and climatic conditions, vineyard location and soil composition, winemaking practices and grape maturity at harvest. The analysis of the phenolic compounds (TPI, tartaric ester and flavonol contents) of the wines indicated that the wines with and without deoxygenation were very similar at all time points during their evolution in bottles (Figure 4), with no significant differences due to the application of sparging (TPI level: *Verdejo* = 0.8940 and *Sauvignon blanc* = 0.0542; tartaric ester level: *Verdejo* = 0.9451 and *Sauvignon blanc* = 0.3198; flavonol level: *Verdejo* = 0.1475 and *Sauvignon blanc* = 0.0499).

Table 1 shows how the DO content significantly affected the phenol content in the *Verdejo* wines, which were the poorest in these compounds. Tartaric esters, which are highly oxidizable compounds, were influenced by the amount of DO present in the *Verdejo* wines, which may be responsible for the increase in color intensity [30]. Despite the fact that sparging was not a determining factor, after 6 months, the *Sauvignon blanc* wines with the highest concentration of dissolved oxygen (W 8.4) showed a lower content of tartaric esters (W 8.4 = 46.9 mg/L) than the corresponding deoxygenated wine (DEOX 8.4 = 48.5 mg/L). This lower content of tartaric esters could be due to oxidation and precipitation of these compounds through the action of oxygen. The flavonols in the *Verdejo* wines, as was the case with the tartaric esters, also increased in the wines with a higher DO, which could affect the brown hues found, as these are responsible for the yellow color of white wines [31]. The length of time in the bottles is the most influential factor affecting the decrease in the levels of these compounds in the wines (Table 1), regardless of the DO content and whether or not sparging was carried out (Figure 4).

#### 3.2.3. Effect on Wine Color

**Figure 5:**
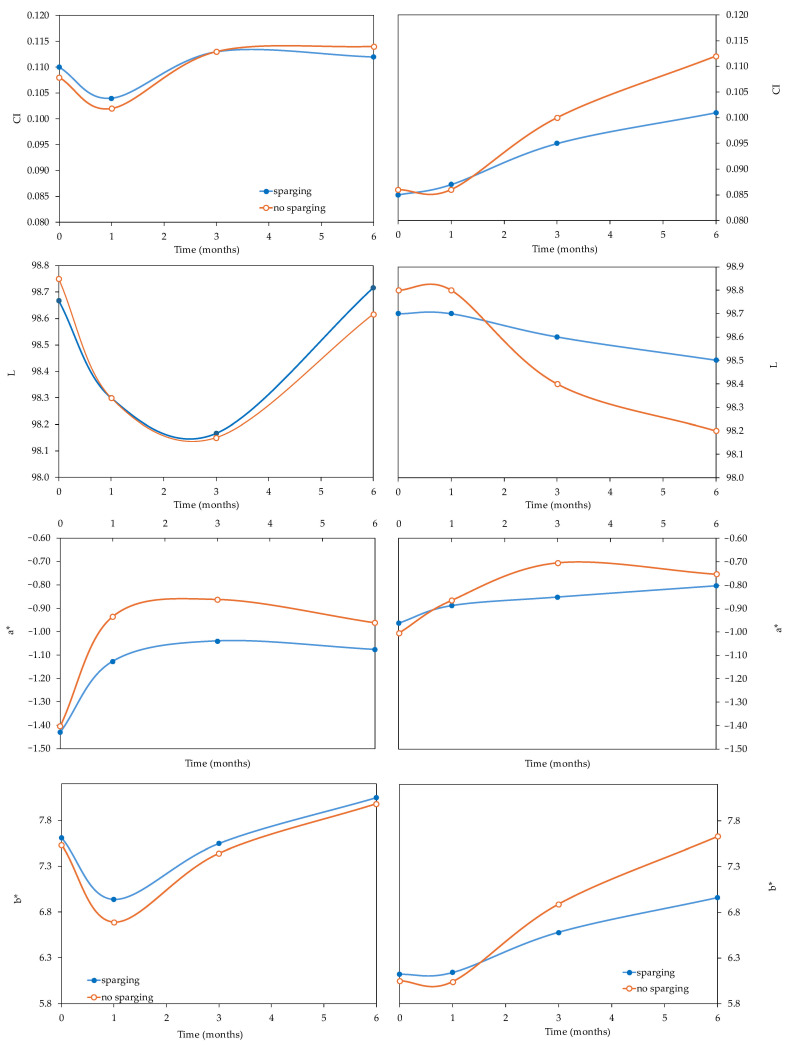
Evolution of the color parameters of *Verdejo* and *Sauvignon blanc* wines with and without sparging treatment over 6 months.

The effects on the color of the wines are shown in Figure 5, including the coloring intensity and the CIELab parameters in the same wines with and without sparging. As shown in Table 1, the MANOVA found that bottle aging time was the factor that affected the color of the wines the most. Sparging and the level of DO in each wine also had an influence on the color parameters, but it depended on the parameter and the variety. However, as described above, initially, the comparison of the wines with and without sparging treatment showed no significant differences in the *Verdejo* and *Sauvignon blanc* wines. Therefore, it can be said that this technique for removing DO from wines before bottling does not immediately alter the color of white wines. However, there were statistically significant differences during bottle aging that could be due to sparging. Thus, during the 6 months in the bottle, an increase in color intensity was observed in the wines of both varieties, which was more evident in the *Sauvignon blanc* wines than in the *Verdejo* wines (Figure 5). The average increase after 6 months in the *Sauvignon blanc* wines, with respect to the value in the just-bottled wine, was 24%, while in the *Verdejo*, it was only 4%, probably because the *Sauvignon blanc* wines had higher tartaric ester and flavonol contents that could have been affected by the action of oxygen. The increase in the color intensity of the *Sauvignon blanc* wines bottled with 3, 6 and 8.4 mg/L of dissolved oxygen was significantly greater (W 3 = 27.5%, W 6 = 29.9% and W 8.4 = 31.9%) than in the deoxygenated wines, which showed increases of 16.9% (DEOX3), 20.3% (DEOX6) and 18.2% (DEOX8.4). Therefore, deoxygenating the *Sauvignon blanc* wines may have contributed to slowing down the browning of the wines, which typically occurs over time in bottles.

The CIELab color parameters also showed differences between the wines during the time in the bottles, especially in the a* (indicates the evolution of the color of the wines between red and green), b* (indicates the evolution of the color between blue and yellow) and luminosity (L) parameters. In the case of the *Verdejo* wines, it was observed that after 6 months in the bottles, the deoxygenated wines with sparging had lower a* values than the corresponding wines with different oxygen levels (W 3, W 6 and W 8.4). Specifically, the a* values in wines W 3, W 6 and W 8.4 increased by 37.7%, 31.5% and 29.2%, respectively, and in wines DEOX 3, DEOX 6 and DEOX 8.4, the increase was 21.4%, 27.2% and 25.2%, respectively. Therefore, the DEOX wines of the *Verdejo* variety showed a lower evolution towards red hues that are indicative of oxidation. That is to say, the removal of oxygen was beneficial in slowing down the development of color during the time spent in the bottles, regardless of whether the oxygen was removed.

In the case of the *Sauvignon blanc* wines, statistically significant differences were found in the CIELab L* and b* parameters at 6 months. Wines W 6 and W 8.4 had higher b* values (greater evolution towards golden tones) and lower luminosity than wines DEOX 6 and DEOX 8.4. In the case of luminosity, the loss during the time in the bottles of wines bottled with and without sparging was very small. On the other hand, the increase in b* values in the wines bottled with oxygen and DEOX wines was greater (W 6 = 27.7% and W 8.4 = 27.6%) than the increase observed in DEOX wines 6 (11.3%) and 8.4 (16.6%). Therefore, it was confirmed that the DEOX 6 could indeed be oxidizing more quickly than the DEOX 8.4.

**Figure 6:**
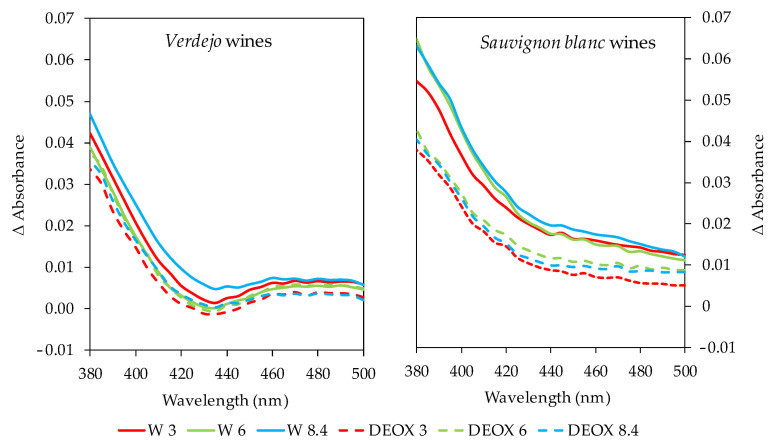
Difference in absorbance spectra of *Verdejo* and *Sauvignon blanc* wines at 6 months versus 0 months of bottle aging.

Figure 6 shows the difference in absorbance in the spectral range between 380 nm and 500 nm of the wines after 6 months in the bottles compared to their spectrum when they were just bottled. In the *Verdejo* wines, and more clearly in the *Sauvignon blanc* wines, it could be seen that the removal of the DO by sparging caused lower absorbance values compared to the corresponding wines bottled with some oxygen. The differences found in this absorbance range indicate that the wines that were deoxygenated had a lower evolution of color towards hues, which are indicative of greater oxidation. Therefore, the removal of the DO by sparging resulted in better maintenance of the color of the wines in the bottles, especially in the *Sauvignon blanc* wines, which had higher of flavonol and tartaric ester contents, which are highly oxidizable compounds and can be altered by the action of oxygen, causing the wines to turn brown and increase their color intensity.

#### 3.2.4. Effect on Volatile Compounds in Wines

- Effect of sparging treatment

**Figure 7:**
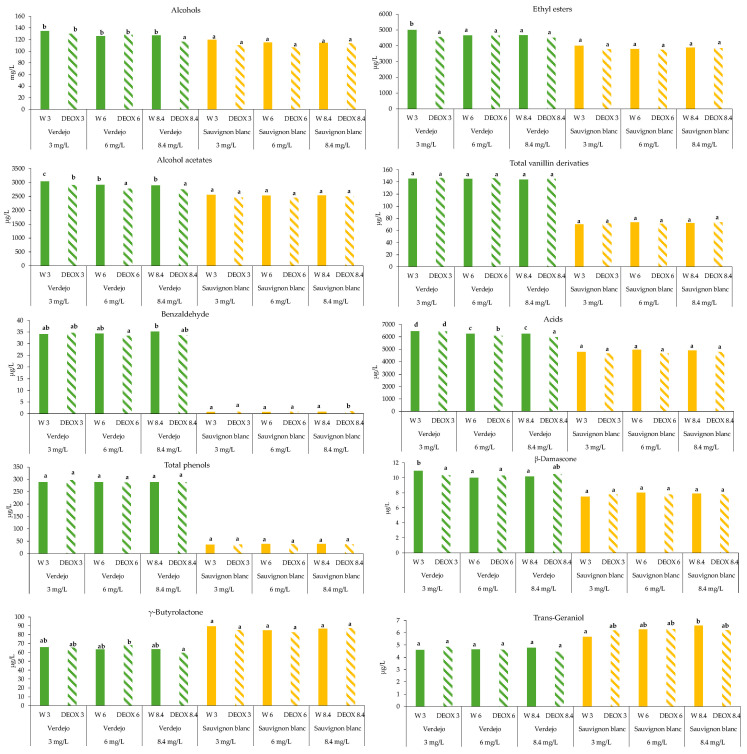
Concentration of the different volatile compounds (grouped as described in Table S2) in *Verdejo* and *Sauvignon blanc* wines after sparging. Different letters indicate statistically significant differences (*p* < 0.05).

The identified volatile compounds were grouped into families in order to discuss the results in a more global way (Figure 7). All the quantified compounds are detailed in Table S2. In addition, the odor threshold and odor active values (OAV = concentration (μg/L)/odor threshold (μg/L)) [2] for each compound are included in Table S3. As mentioned in the literature, only compounds with an OAV > 1 can be perceived by the human nose and have a real impact on olfactory perception [2,32].

Sparging affected some groups of compounds in the wines but it depended on the variety studied. Thus, the deoxygenation process using sparging with N_2_ only affected the composition of some families of volatile compounds of sensory interest at the time of bottling. In the case of the *Verdejo* wines, an effect was seen on acetate esters and volatile acids. In general, the wines that were deoxygenated (DEOX 3, 6 and 8.4) had lower of acetate ester contents (4.3%, 4.7% and 4.2%) compared to the corresponding non-deoxygenated wine (W 3, 6 and 8.4), probably due to a carry-over of these compounds during the sparging process. These differences were mainly due to the effect on isoamyl acetate, which was the main compound in this group and, moreover, is the most volatile of all the compounds analyzed in this group. Therefore, it could be more easily altered by the carry-over of the DO with N_2_ during sparging. This compound brings fruity notes of banana to wines [32,33,34,35]. In all the *Verdejo* wines, this compound was found at concentrations above the perception threshold (30 µg/L) and the OAV was higher than 1; therefore, it can be said that the sparging process to remove 3, 6 and 8.4 mg/L of DO in the wines resulted in a significant loss in the concentration of this compound of sensory interest. In the case of volatile fatty acids, differences were seen for the wines with DO concentrations of 6 and 8.4 mg/L, with a loss percentage of 2.8% for DEOX 6 and 4.2% for DEOX 8.4. These differences were mainly due to the differences in hexanoic, octanoic and decanoic acid levels, which were the most abundant volatile fatty acids. According to the consulted studies, these compounds contribute notes of rancid cheese [33,34,36]. In this case, only octanoic acid was found at concentrations above the perception threshold (500 µg/L). However, the consulted studies indicated that, as a whole, when the concentration of fatty acids is below 10 mg/L, they tend to give pleasant notes to wines. On the other hand, when their concentration is above this value, unpleasant odors of cheese, rancidity and animal odors begin to appear [33,34]. In this study, all the wines had a concentration below the threshold of 10 mg/L; therefore, a loss of these compounds could have produced a loss of positive aromatic complexity. Furthermore, this compound had an OAV > 1 and therefore, it could be perceived sensorially. The DEOX 3 wine showed a 5% lower β-damascenone content than the W 3 wine with oxygen, with no differences found with the rest of the wines. This result was surprising, as one would expect that when a greater amount of N_2_ is applied to remove oxygen, there would be a greater loss due to drag-out with the N_2_. β-damascenone contributes baked apple, floral and honey notes when its concentration is above 0.05 µg/L [33,34]. All the wines studied had concentrations that exceeded this threshold and their OAVs were higher than 1; therefore, sparging could have had a negative effect on the DEOX 3 wines. There were no effects on the other families of volatile compounds in the *Verdejo* wine.

In the case of the *Sauvignon blanc* wines, sparging had no effect on any of the groups of compounds analyzed, except in the case of benzaldehyde, which is known to contribute notes of bitter almonds [33,37]. DEOX 8.4 had a higher benzaldehyde content than the other wines, but this did not have an impact on the aroma since its content was well below the perception threshold (2000 µg/L) [33]. Sparging and the oxygen content did not show clear effects on the trans-geraniol content.

- Effect of time in bottles

**Figure 8:**
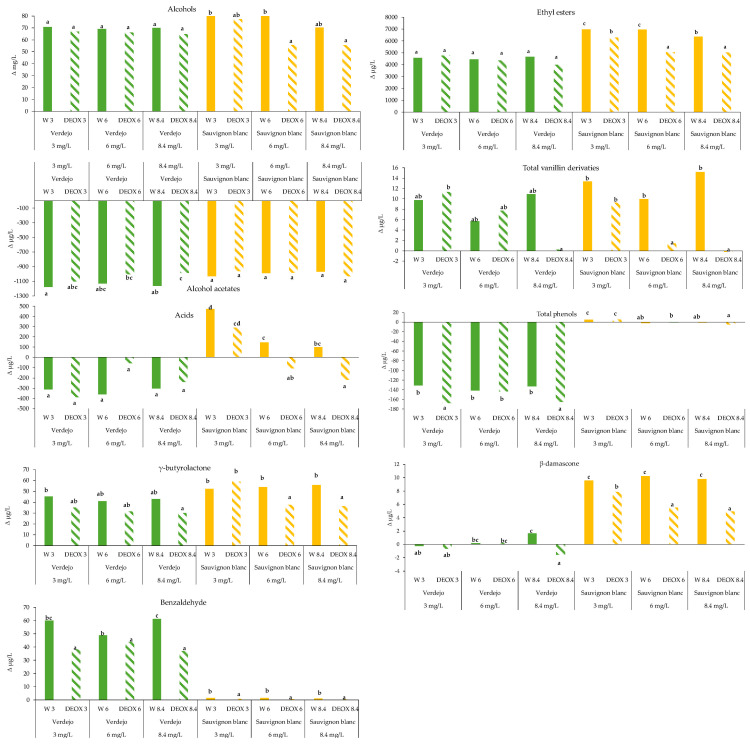
Gain or loss (in concentration) of the different groups of volatile compounds studied at the end of bottle aging in *Verdejo* and *Sauvignon blanc* wines. Different letters indicate statistically significant differences (*p* < 0.05).

Figure 8 shows the variation in the concentration of the different groups of compounds in the wines with different DO concentrations and deoxygenated wines between 6 months and 0 months in the bottles (after sparging). The changes in the levels of these compounds are detailed in Table S4. In the *Verdejo* wines, increases in the alcohol and ethyl ester contents were observed, with no statistical differences between the wines with some level of oxygen and the corresponding deoxygenated wine. Therefore, this group of compounds evolved over time in the bottles in the same way in the wines with oxygen and in the DEOX wines. In the *Sauvignon blanc* wines, there was also an increase in the alcohol and ethyl ester contents with bottle age and, in this case, there were significant differences between the wines that were bottled with some concentration of oxygen and the corresponding DEOX wine. Thus, in general, the wines that had been bottled with oxygen had a higher content of these groups of compounds than the deoxygenated wines. These differences were clearer for the ethyl esters, mainly due to an increase in the content of monoethyl and diethyl succinate. These two ethyl esters increased the most in both the *Verdejo* and the *Sauvignon blanc* wines.

According to the literature, the content of ethyl esters can decrease, remain constant or increase depending on the storage conditions [38,39]. Increases are usually due to esterification reactions with acids and alcohols, producing an increase in the levels of mono- and dicarboxylic ethyl esters of other acids, such as monoethyl and diethyl succinate [38,40,41], similar to the results obtained in this study (Table S3). In general, in the *Sauvignon blanc* wines, the wines that had more oxygen at the time of bottling (W 8.4) showed a smaller increase in alcohols, especially 2-phenylethanol, and esters, especially the esters that increase over time (monoethyl, ethyl lactate and diethyl succinate) (Table S4). In general, ethyl esters contribute fruity notes to wines [32,33,34,35]. Despite the increase in some esters in both the *Verdejo* and *Sauvignon blanc* wines, there was a decrease in the ethyl hexanoate, octanoate and decanoate contents (with larger increases in the *Verdejo* than in the *Sauvignon blanc*), which is in accordance with the results of [39]. In the *Verdejo* wines, deoxygenation caused the esters that decrease to decrease less compared to when sparging had not been carried out; thus, the removal of oxygen reduced these losses during the time in the bottles. As mentioned above, the acetates of alcohols, mainly isoamyl acetate, are also known to contribute fruity notes of banana [32,33,34,35]. In both the *Verdejo* and *Sauvignon blanc* wines, there was a loss of these compounds during the evolution of the wines in the bottles. These results are in accordance with those of [38,39,40], which indicates that storage in bottles results in a loss of this type of compound due to hydrolysis reactions. Significant differences were only found in the *Verdejo* wines. Thus, for the wines with the highest concentration of oxygen (W 8.4), the deoxygenation process (DEOX 8.4) caused the loss of these compounds to be 15.2% lower, and the DEOX 8.4 wines were able to retain a higher concentration of these compounds, including isoamyl acetate, which is responsible for fruity aromas.

The volatile fatty acids evolved differently in the two varieties. In the *Verdejo* wines, there was a loss of these compounds during the time in the bottles, while in the *Sauvignon blanc* wines, there was an increase, except in the DEOX 6 and DEOX 8.4 wines, which showed a significant loss of these compounds. Some previous studies observed a loss of these compounds [41,42] and others found an increase after storage in bottles, which was attributed to the conversion of low-chain saturation fatty acids to high-chain saturation fatty acids during aging [40,43]. Significant differences were only found in the *Sauvignon blanc* wines, which showed decreasing contents with increasing oxygen (W3 > W6 > W8.4) and a lower content when sparging had been carried out: the oxygenated wines (W3, W6 and W8.4) had higher fatty acid contents than the corresponding deoxygenated wines (DEOX 3, DEOX 6 and DEOX 8.4). As mentioned above, these compounds contribute negative aromas to wines when their content is above 10 mg/L. On the other hand, if their content is below 10 mg/L, these compounds can contribute to improving the complexity of wines. In the *Verdejo* wines, the volatile fatty acid content was below this concentration. Therefore, the removal of high concentrations of oxygen from the wines could, to some extent, reduce the increase in the levels of these compounds, decreasing the complexity of the wines.

The vanillin derivative content increased over time in the bottles except for the *Sauvignon blanc* wine DEOX 8.4. In the *Verdejo* and *Sauvignon blanc* wines that had been sparged, greater increases in the vanillin derivative content were found when less oxygen had been removed (DEOX 3 > DEOX 6 > DEOX 8.4). In the *Sauvignon blanc* wines, there were also differences between the deoxygenated and non-deoxygenated wines, with a generally greater increase observed in the wines not subjected to oxygen removal than in the deoxygenated wines. These compounds are aldehydes and they contribute vanilla notes [44,45]. However, the vanillin derivative content was well below the perception threshold (3000 and 1000 µg/L) as was expected since these compounds are usually found in high concentrations in wines aged in wood, and this is not the case with these wines. Therefore, from a sensory point of view, it did not have a negative effect. The benzaldehyde content increased, especially in the *Verdejo* wines, and the increase was significantly lower in the DEOX wines than in the oxygenated wines. The same results were found for the *Sauvignon blanc* wines, but the increase over time in the bottles was much lower than in the case of the *Verdejo* wines. As with the vanillin derivatives, the benzaldehyde content was found to be well below the perception threshold (2000 µg/L) as benzaldehyde is typically found in wines aged in wood.

In general, the γ-butyrolactone content increased during the time in the bottles, with the deoxygenated *Verdejo* and *Sauvignon blanc* wines showing a smaller increase than those that were bottled without deoxygenation (Figure 8). In a literature review [39], the authors indicate that research on the changes in the levels of some lactones during the bottle storage process is limited or controversial, and the alteration mechanisms are not clear since in some cases, their content increases and in others, they remain stable. These compounds contribute caramel, sweet and fruity notes [46], and they were found in all the wines at concentrations above the perception threshold (35 µg/L). The aromas of the deoxygenated wines may have a lower contribution from these aroma notes. In general, β-damascenone levels also increased over time in the bottles, which align with the results in the literature [47]. The deoxygenated *Sauvignon blanc* wines showed a smaller increase in the levels of this compound; this trend was also observed in the *Verdejo* wines that had the highest oxygen levels (W 8.4) compared to the corresponding deoxygenated wine (DEOX 8.4). β-Damascenone contributes apple fruit, sweet and honey notes [32,33]. Therefore, especially in *Sauvignon blanc*, sparging could affect these attributes of the final wine.

The content of volatile phenols evolved differently in the *Verdejo* wines and the *Sauvignon blanc* wines. In the former, their contents decreased, while in the latter, depending on the treatment, the contents increased, decreased or remained stable. According to different bottle aging studies, the contents of these compounds increase or decrease depending on different factors [48,49]. The *Verdejo* wines that had been subjected to sparging had a greater decrease in the volatile phenol content than wines with oxygen (mainly DEOX 3 and DEOX 8.4). On the other hand, no differences were found between the oxygenated and deoxygenated *Sauvignon blanc* wines. These compounds provide notes of clove, spices and cinnamon [50].

### 3.3. Effect on the Sensory Profile of the Wines

**Figure 9:**
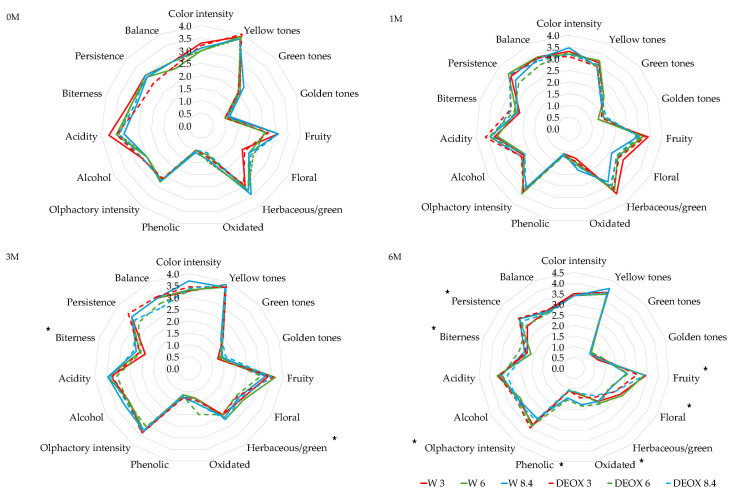
Sensory analysis of *Verdejo* wines after sparging (0 M) and during bottle aging (1 M, 3 M and 6 M). * indicates statistically significant differences (*p* < 0.05) between the wines.

**Figure 10:**
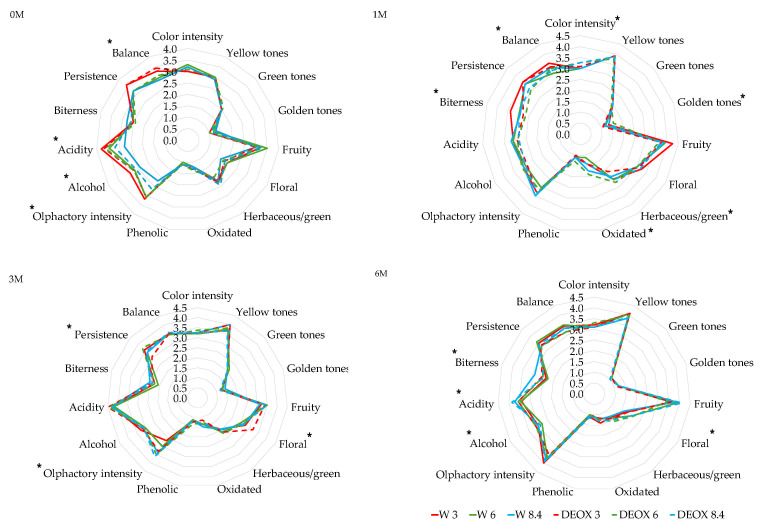
Sensory analysis of *Sauvignon blanc* wines after sparging (0 M) and during bottle aging (1 M, 3 M and 6 M). The asterisk (*) indicates statistically significant differences (*p* < 0.05) between the wines.

The results of the sensory analysis of the *Verdejo* and *Sauvignon blanc* wines is shown in Figure 9 and Figure 10, respectively. The results show that before bottling, there were no differences between the non-deoxygenated and deoxygenated wines. During the first months in the bottles, there were still no significant differences. After 6 months in the bottles, significant differences were found, mainly in the olfactory phase. In general, the *Verdejo* wines with 6 and 8.4 mg/L of dissolved oxygen removed (DEOX 6 and DEOX 8.4) showed more fruity aromas, which are typically produced by ethyl esters and acetates of alcohols, and less oxidized and phenolated odors, which are due to the formation of oxidation aldehydes such as strecker aldehydes and the formation of volatile phenols, than those bottled with 6 and 8.4 mg/L of dissolved oxygen (W 6 and W 8.4). The analysis of the volatile compounds indicated a greater increase in compounds responsible for fruity aromas in the wines that were bottled with different DO levels, which could have been formed by esterification reactions. Therefore, it was expected that the tasting panel would perceive these wines as having more fruity notes, but the opposite was true. This could be due to an increase in the formation of aldehydes through the action of oxygen that could mask the fruity aromas. Therefore, the evolution of the DEOX 6 and DEOX 8.4 wines after 6 months in the bottles was better than that of the corresponding wines bottled with 6 and 8.4 mg/L oxygen, possibly due to the presence of N_2_, which prevents the formation of oxidation aldehydes.

As for the *Sauvignon blanc* wines, after sparging, differences were found between some wines before bottling, mainly in olfactory intensity and in taste parameters such as alcohol, acidity and balance. These differences were mainly due to the oxygen level rather than to the fact that the wines were deoxygenated. With the *Verdejo* wines, initially, the sparging process did not seem to affect the sensory profile of the wines. After the first month in the bottles, although there were statistically significant differences in parameters such as color intensity, golden tones, herbaceous aromas, oxidized odors, bitterness and balance between the different wines, there were no differences between W 3 vs. DEOX 3, W 6 vs. DEOX 6 or W 8.4 vs. DEOX 8.4 in terms of the visual, olfactory or taste phase. At the end of the aging time in the bottles, significant differences were found in the fruity aroma and in the taste parameters of alcohol taste, acidity and bitterness between the different wines. However, differences due to sparging were only seen in the W 8.4 and DEOX 8.4 wines, with the fruit aroma being stronger in the deoxygenated wine compared to the wine bottled with 8.4 mg/L oxygen. In the *Verdejo* wines, the analysis of volatile compounds showed that there a larger increase in the compounds responsible for fruity aromas (ethyl esters) in the wines bottled with 8.4 mg/L oxygen than in the deoxygenated wines, which probably formed through esterification processes. Therefore, one would expect stronger fruity aromas in the oxygenated wines. On the other hand, in the oxygenated wines, oxidation aldehydes could be forming, masking the fruity aromas and diminishing their intensity in the olfactory phase of the tasting. Therefore, sparging wines using N_2_ with high amounts of oxygen could be beneficial in preserving or not masking fruit aromas during bottle aging, preventing the formation of oxidation compounds such as aldehydes. The other olfactory and taste parameters did not show differences between W 3 vs. DEOX 3, W 6 vs. DEOX 6 and W 8.4 vs. DEOX 8.4.

### 3.4. Multivariate Statistical Analysis

**Figure 11:**
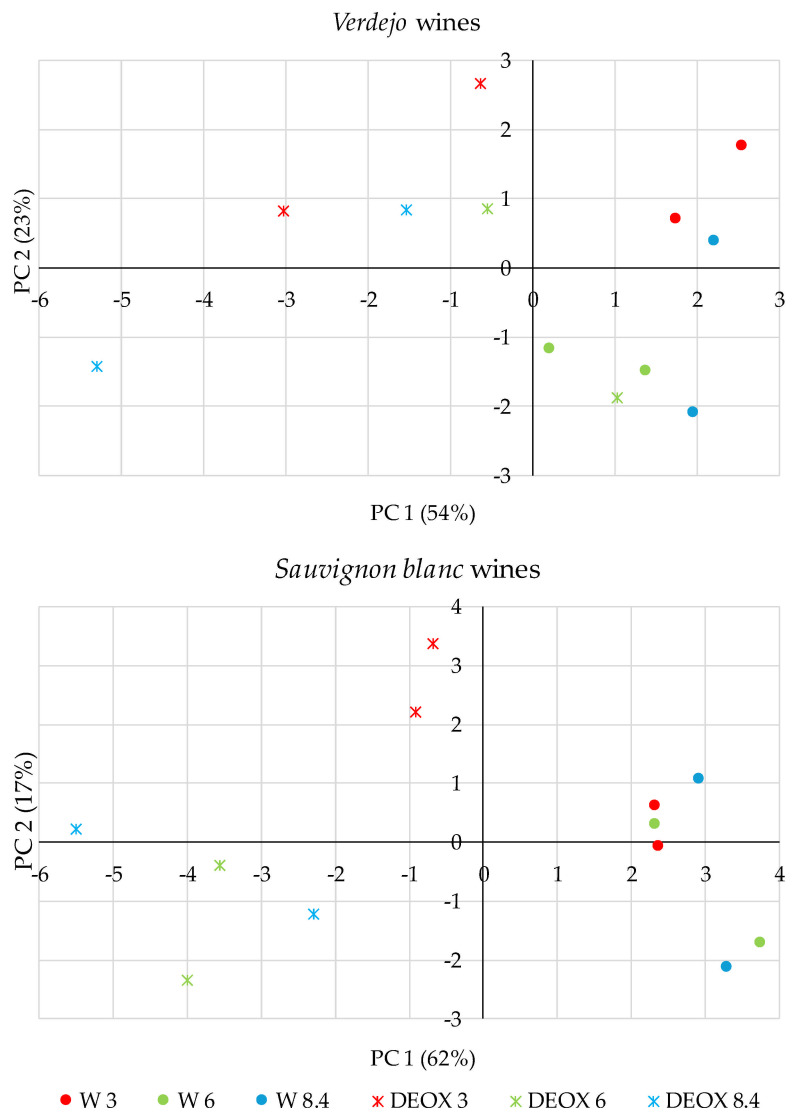
Principal component analysis (PCA) of *Verdejo* and *Sauvignon blanc* wines after 6 months in bottles (6 M).

Knowing the chemical parameters that varied the most in the wines during their time in the bottles, and in order to extract the variables that define the wines with and without sparging treatment after 6 months in the bottles, a principal component analysis (PCA) was performed. In the case of the *Verdejo* wines, the first two components explained 77% of the total variance (Table 2) (Figure 11). In general, it could be observed that PC1, which explained 54% of the total variance, could be used to differentiate between the wines that were bottled with dissolved oxygen (positive side of the plane) from those that had been deoxygenated by sparging with N_2_ (negative side of the plane) except for the wines that were bottled with 6 mg/L DO. As can be seen in Table 2, the F-SO_2_ and T-SO_2_ contents and CIELab L* parameter were highly associated with DEOX wines since these wines were better protected against oxidation and had higher brightness due to lower color evolution during bottle aging. In contrast, the levels of most of the volatile compounds, the color intensity and the CIELab a* parameter were more associated with wines bottled with different oxygen levels. This was because these wines reached the end of bottle aging with a higher volatile compound content and a greater color evolution due to the action of oxygen. Therefore, sparging allowed the *Verdejo* wines to maintain higher levels of free and total sulfur dioxide and to maintain a better color after 6 months in the bottles. On the other hand, these wines had a lower volatile compound content.

In the case of the *Sauvignon blanc* wines, the first 2 components from the PCA explained 79% of the total variance. Thus, PC1, which explained 62% of the variance, allowed for a good separation of the wines that had some oxygen (positive part of the plane) and those that had oxygen removed by sparging (negative part of the plane). Thus, as with the *Verdejo* wines, many of the volatile compounds studied were positively correlated with the wines with different oxygen concentrations. That is, at the end of the time in the bottles, the wines with some oxygen had, in general, a greater increase in these compounds than the deoxygenated wines but there were no differences between the wines with different oxygen levels.

Therefore, we confirmed that the wines with different oxygen levels showed higher contents of most of the volatile compounds studied in this work compared to those that were deoxygenated. In addition, the wines with different oxygen levels had a higher total acidity and a greater color evolution due to a greater increase in color intensity and CIELab a* and b* parameters. On the other hand, the deoxygenated *Verdejo* wines reached the end of aging with higher levels of F-SO_2_ and T-SO_2_ and L* values. Therefore, sparging allowed the wines to be protected during bottle aging with better preservation of their color.

## 4. Conclusions

The amount of nitrogen required to reduce the oxygen concentration in wines to a level of 0.3 mg/L increases proportionally with the oxygen concentration. The *Verdejo* wines required more N_2_ than the *Sauvignon blanc* wines to remove concentrations of 3, 6 and 8.4 mg/L (between 10% and 25% depending on the oxygen level).

The changes in the wines during bottle aging were mainly time-dependent, and sparging did not affect the basic chemical parameters of the *Verdejo* and *Sauvignon blanc* wines in the same way. As expected, F-SO_2_ levels decreased significantly in the wines with time in the bottles, especially in wines bottled with higher oxygen levels. Therefore, oxygen removal through sparging allowed for better preservation of F-SO_2_ and T-SO_2_ levels, thus preserving the *Verdejo* and *Sauvignon blanc* wines better.

The removal of dissolved oxygen (DO) through sparging did not significantly affect the phenolic compound content. However, in the absence of sparging, the presence of DO in the wine influenced the evolution of tartaric esters in the *Sauvignon blanc* wines during bottle aging.

The deoxygenation process did not affect the color of the wines in the short term, but during bottle aging, the removal of DO by sparging had a protective effect on color evolution, which was more evident in the *Sauvignon blanc* wines. It reduced the tendency to develop reddish tones and prevented an increase in the color intensity compared to the wines bottled with different oxygen levels.

Sparging affected the volatile compound composition, mainly in the *Verdejo* wines, resulting in decreases in the concentrations of acetate esters and acids, which are responsible for providing fruity notes and complexity, respectively. Moreover, in wines bottled after sparging, and especially the *Sauvignon blanc* wines, the increase in the levels of volatile compounds, such as alcohols, ethyl esters, acids, vanillin derivatives, γ-butyrolactone, β-damascenone and benzaldehyde, during the time in the bottles were smaller.

Sensorially, sparging did not cause significant differences in the *Verdejo* and *Sauvignon blanc* wines, but with increasing bottling time, it was observed that all the deoxygenated wines improved the olfactory profile, especially when the oxygen concentrations removed were high.

This study represents a valuable line of research for the future. However, the degree of sparging and the bottles could be limiting factors but the extrapolation to 0.75 L bottles and natural cork closures should be approached with caution. Differences in bottle volume and stopper type can significantly influence oxygen incorporation and wine evolution. For these reasons, studies involving extended storage times (e.g., 12 months or more), larger volume bottles and different closure systems would help to better understand the stability and aging potential of white wines under commercial conditions.

## Figures and Tables

**Table 1:** Multivariate analysis of variance (MANOVA) of chemical parameters in *Verdejo* and *Sauvignon blanc* wines.

	*Verdejo* Wines			*Sauvignon blanc* Wines		
	O_2_ Level	Sparging	Time	O_2_ Level	Sparging	Time
pH	0.3041	**0.0003**	**0.0000**	0.3653	**0.0463**	**0.0000**
Total acidity (g/L)	0.5591	**0.0106**	**0.0197**	0.8243	**0.0000**	**0.0000**
Alcoholic content (% *v*/*v*)	0.6357	0.1341	0.0890	0.4025	**0.0238**	**0.0032**
Volatile acidity (g/L)	**0.0039**	**0.0390**	**0.0003**	0.4285	0.6178	**0.0000**
F-SO_2_ content (mg/L)	**0.0000**	0.3513	**0.0000**	**0.0062**	**0.0000**	**0.0000**
T-SO_2_ content (mg/L)	**0.0000**	**0.0139**	**0.0000**	0.1883	**0.0000**	**0.0000**
TPI	0.3715	0.8940	**0.0000**	0.8572	0.0542	**0.0000**
Tartaric ester content (mg/L)	**0.0189**	0.9451	**0.0000**	0.4177	0.3198	**0.0000**
Flavonol content (mg/L)	**0.0039**	0.1475	**0.0000**	0.2629	0.0499	**0.0000**
Color intensity	**0.0057**	0.3551	**0.0000**	**0.0091**	**0.0000**	**0.0000**
L*	**0.0004**	0.8941	**0.0001**	0.9145	0.3167	**0.0015**
a*	**0.0275**	**0.0006**	**0.0000**	0.4296	0.0679	**0.0000**
b*	0.7542	**0.0484**	**0.0000**	0.1327	**0.0011**	**0.0000**
Total alcohol content (mg/L)	0.0815	0.8589	**0.0000**	**0.0053**	0.0757	**0.0000**
Total ethyl ester content (µg/L)	**0.0220**	0.3871	**0.0000**	**0.0000**	**0.0000**	**0.0001**
Total acetate ester content (µg/L)	**0.0045**	0.6331	**0.0000**	**0.0000**	**0.0000**	0.1042
Total fatty acid content (µg/L)	0.2607	0.5942	**0.0018**	**0.0373**	0.0184	**0.0054**
Total vanillic derivative content (µg/L)	0.8128	0.0755	**0.0000**	**0.0253**	**0.0008**	**0.0000**
Benzaldehyde content (µg/L)	0.0956	**0.0003**	**0.0000**	0.6656	**0.0227**	**0.0000**
γ-butyrolactone content (µg/L)	**0.0035**	0.6617	**0.0000**	0.8329	**0.0250**	**0.0000**
β-damascenone content (µg/L)	0.9014	0.4556	**0.0000**	0.2904	**0.0000**	**0.0000**
Total terpene content (µg/L)	0.0811	**0.0003**	**0.0000**	0.3388	0.8668	**0.0000**
Total phenol content (µg/L)	0.3927	0.9556	**0.0000**	**0.0010**	0.9571	**0.0034**

Bolded values indicate statistically significant differences (*p* < 0.005).

**Table 2:** Loading values of the variables used in PCA for *Verdejo* and *Sauvignon blanc* wines after six months in bottles.

	*Verdejo* Wines		*Sauvignon blanc* Wines	
	PC1	PC2	PC1	PC2
pH			−0.33	0.52
Total acidity			0.82	−0.48
Alcoholic strength			−0.63	−0.46
F-SO_2_ content	−0.81	0.06	−0.77	0.46
F-SO_2_ content	−0.73	0.57	−0.76	0.54
Color intensity	0.64	−0.58	0.85	−0.41
Tartaric ester content			0.47	0.56
Flavonol content			0.69	0.24
L*	−0.58	0.62	−0.82	0.43
a*	0.83	−0.2	0.23	−0.38
b*			0.81	−0.35
Total alcohol content			0.82	0.41
Total alcohol acetate content	0.37	0.65		
Total ethyl ester content			0.9	0.31
Total acid content	0.66	0.63	0.89	0.36
Total vanillic derivative content	0.89	0.04	0.92	0.21
Benzaldehyde content	0.48	0.61	0.89	−0.04
γ-butyrolactone content	0.86	0.36	0.74	0.63
β-damascenone content	0.79	0.09	0.97	0.13

## Data Availability

The original contributions presented in the study are included in the article/Supplementary Material, further inquiries can be directed to the corresponding authors.

## Supplementary Materials

The following supporting information can be downloaded at: https://www.mdpi.com/article/10.3390/foods14132272/s1, Table S1: Classic enological parameters of different *Verdejo* and *Sauvignon blanc* wines after sparging (0 M) and during aging in bottle (1 M, 3 M and 6 M); Table S2: Concentration of volatile compounds in the different *Verdejo* and *Sauvignon blanc* wines; Table S3: Odor threshold and odor active values (OAVs) of each volatile compound of the different *Verdejo* and *Sauvignon blanc* wines studied; Table S4: Increasing or decreasing concentration (µg/L) of volatile compounds during the aging in bottle of the different *Verdejo* and *Sauvignon blanc* wines studied.
