# Supplementary material for: Impact of Nitrogen Sparging on Chemical and Sensory Characteristics of Verdejo and Sauvignon blanc Wines
Source: Foods. 2025 Jun 26;14(13):2272. doi: 10.3390/foods14132272 (PMC12248951; doi:10.3390/foods14132272)
Supplement: Supplementary file 1 [file foods-14-02272-s001.zip › foods-3669261-supplementary.pdf]

Table S1. Classic enological parameters of different *Verdejo* and *Sauvignon blanc* wines studied after sparging (0M) and during aging in bottle (1M, 3M and 6M)

	***Verdejo* wines**						***Sauvignon blanc* wines**					
**0M**	**W 3**	**DEOX 3**	**W 6**	**DEOX 6**	**W 8.4**	**DEOX 8.4**	**W 3**	**DEOX 3**	**W 6**	**DEOX 6**	**W 8.4**	**DEOX 8.4**
**pH**	3.22 ± 0.01 a	3.24 ± 0.01 bc	3.22 ± 0.00 ab	3.24 ± 0.00 c	3.23 ± 0.00 abc	3.25 ± 0.01 c	3.07 ± 0.02 a	3.08 ± 0.01 a	3.07 ± 0.02 a	3.06 ± 0.03 a	3.07 ± 0.01 a	3.06 ± 0.01 a
**Total acidity (g/L)**	5.6 ± 0.0 c	5.5 ± 0.0 b	5.55 ± 0.1 bc	5.4 ± 0.0 a	5.5 ± 0.0 b	5.4 ± 0.0 a	6.85 ± 0.1 ab	6.8 ± 0.0 a	6.8 ± 0.0 a	6.8 ± 0.0 a	6.8 ± 0.0 a	6.8 ± 0.0 a
**Ethanol content (% vol)**	12.80 ± 0.07 a	12.80 ± 0.07 a	12.75 ± 0.07a	12.65 ± 0.05 a	12.75 ± 0.07 a	12.60 ± 0.07 a	13.60 ± 0.02 a	13.60 ± 0.03 a	13.60 ± 0.03 a	13.60 ± 0.02 a	13.60 ± 0.02 a	13.60 ± 0.03 a
**Volatile acidity (g/L)**	0.31 ± 0.01 a	0.34 ± 0.01 b	0.31 ± 0.01 a	0.35 ± 0.01 b	0.32 ± 0.01 a	0.35 ± 0.01 b	0.37 ± 0.01 a	0.37 ± 0.01 a	0.38 ± 0.04 a	0.37 ± 0.03 a	0.38 ± 0.01 a	0.40 ± 0.03 a
**F-SO_2 (mg/L)**	26 ± 0 b	25 ± 1 ab	26 ± 1 ab	25 ± 0 ab	24 ± 0 a	24. ± 0 a	44 ± 0 a	42 ± 2 a	43 ± 1 a	43 ± 0 a	44 ± 0 a	44 ± 0 a
**T-SO_2 (mg/L)**	99 ± 0 d	98 ± 0 c	95 ± 0 b	94 ± 0 a	94 ± 1 a	95 ± 0 b	101 ± 0 b	95 ± 3 ab	92 ± 5 a	98 ± 1 ab	96 ± 2 ab	92 ± 3 a
**1M**	**W 3**	**DEOX 3**	**W 6**	**DEOX 6**	**W 8.4**	**DEOX 8.4**	**W 3**	**DEOX 3**	**W 6**	**DEOX 6**	**W 8.4**	**DEOX 8.4**
**pH**	3.25 ± 0.02 a	3.23 ± 0.01 a	3.25 ± 0.01 a	3.24 ± 0.02 a	3.25 ± 0.01 a	3.25 ± 0.01 a	3.16 ± 0.01 ab	3.23 ± 0.02 b	3.14 ± 0.01 a	3.22 ± 0.02 b	3.23 ± 0.02 b	3.22 ± 0.06 b
**Total acidity (g/L)**	5.6 ± 0.0 d	5.5 ± 0.0 bc	5.5 ± 0.0 c	5.4 ± 0.0 b	5.5 ± 0.0 c	5.4 ± 0.0 a	6.7 ± 0.0 b	6.6 ± 0.1 ab	6.6 ± 0.0 ab	6.6 ± 0.0 ab	6.6 ± 0.1 ab	6.6 ± 0.1 a
**Ethanol content (% vol)**	12.50 ± 0.02 a	12.55 ± 0.03 a	12.55 ± 0.02 a	12.5 ± 0.07 a	12.55 ± 0.04 a	12.5 ± 0.07 a	13.5 ± 0.00 a	13.55 ± 0.07 a	13.5 ± 0.00 a	13.6 ± 0.00 a	13.5 ± 0.00 a	13.5 ± 0.00 a
**Volatile acidity (g/L)**	0.30 ± 0.00 a	0.32 ± 0.01 a	0.31 ± 0.01 a	0.30 ± 0.01 a	0.31 ± 0.01 a	0.33 ± 0.01 a	0.38 ± 0.01 a	0.41 ± 0.00 a	0.43 ± 0.04 a	0.40 ± 0.03 a	0.41 ± 0.02 a	0.43 ± 0.03 a
**F-SO_2 (mg/L)**	15 ± 0 b	12 ± 0 a	14 ± 0 b	13 ± 1 a	12 ± 1 a	14 ± 1 b	20 ± 3 bc	25 ± 2 c	14 ± 2 a	21 ± 3 bc	16 ± 0 ab	22 ± 0 c
**T-SO_2 (mg/L)**	90 ± 1 bc	92 ± 2 bc	90 ± 1 b	92 ± 1 bc	82 ± 2 a	93 ± 0 c	63 ± 1 a	79 ± 2 d	65 ± 0 a	74 ± 1 c	70 ± 0 b	79 ± 1 d
**3M**	**W 3**	**DEOX 3**	**W 6**	**DEOX 6**	**W 8.4**	**DEOX 8.4**	**W 3**	**DEOX 3**	**W 6**	**DEOX 6**	**W 8.4**	**DEOX 8.4**
**pH**	3.19 ± 0.04 d	3.05 ± 0.06 abc	3.14 ± 0.05 cd	3.03 ± 0.01 ab	3.12 ± 0.05 bcd	3.01 ± 0.00 a	3.11 ± 0.02 a	3.10 ± 0.01 a	3.09 ± 0.01 a	3.10 ± 0.01 a	3.09 ± 0.01 a	3.09 ± 0.01 a
**Total acidity (g/L)**	5.6 ± 0.02 b	5.5 ± 0.02 a	5.6 ± 0.05 b	5.6 ± 0.00 ab	5.5 ± 0.01 ab	5.6 ± 0.00 b	6.7 ± 0.01 c	6.5 ± 0.03 a	6.6 ± 0.03 bc	6.6 ± 0.04 ab	6.7 ± 0.07 c	6.6 ± 0.03 bc
**Ethanol content (% vol)**	12.55 ± 0.01 a	12.50 ± 0.03 a	12.55 ± 0.05 a	12.55 ± 0.02 a	12.55 ± 0.05 a	12.55 ± 0.05 a	13.70 ± 0.00 a	13.70 ± 0.00 a	13.70 ± 0.00 a	13.70 ± 0.00 a	13.70 ± 0.00 a	13.70 ± 0.00 a
**Volatile acidity (g/L)**	0.35 ± 0.01 a	0.35 ± 0.03 a	0.34 ± 0.01 a	0.35 ± 0.03 a	0.36 ± 0.01 a	0.35 ± 0.01 a	0.40 ± 0.01 a	0.41 ± 0.01 a	0.42 ± 0.02 bc	0.41 ± 0.01 a	0.42 ± 0.01 c	0.40 ± 0.02 a
**F-SO_2 (mg/L)**	10 ± 1.27 bc	11 ± 0.52 c	8 ± 0.17 ab	10 ± 0.17 bc	8 ± 0.47 a	9 ± 0.20 b	14 ± 0.03 c	15 ± 0.88 cd	9 ± 0.98 b	20 ± 0.27 e	6 ± 1.27 a	17 ± 1.35 d
**T-SO_2 (mg/L)**	79 ± 1 d	73 ± 1 c	70 ± 2 c	61 ± 3 b	56 ± 0 a	79 ± 2 d	66 ± 1 ab	73 ± 1 b	57 ± 3 a	72 ± 5 b	58 ± 8 a	68 ± 5 ab
**6M**	**W 3**	**DEOX 3**	**W 6**	**DEOX 6**	**W 8.4**	**DEOX 8.4**	**W 3**	**DEOX 3**	**W 6**	**DEOX 6**	**W 8.4**	**DEOX 8.4**
**pH**	3.24 ± 0.01 b	3.25 ± 0.03 b	3.25 ± 0.01 b	3.19 ± 0.02 a	3.25 ± 0.01 b	3.24 ± 0.00 b	3.15 ± 0.01 a	3.18 ± 0.02 a	3.17 ± 0.02 a	3.16 ± 0.01 a	3.16 ± 0.02 a	3.17 ± 0.01 a
**Total acidity (g/L)**	5.5 ± 0.0 a	5.5 ± 0.1 a	5.5 ± 0.1 a	5.6 ± 0.2 a	5.5 ± 0.0 a	5.6 ± 0.0 a	6.8 ± 0.0 abc	6.7 ± 0.1 a	6.8 ± 0.1 bc	6.7 ± 0.0 abc	6.9 ± 0.0 c	6.7 ± 0.1 ab
**Ethanol content (% vol)**	12.48 ± 0.11 a	12.55 ± 0.00 a	12.48 ± 0.11 a	12.55 ± 0.00 a	12.55 ± 0.00 a	12.48 ± 0.11 a	13.55 ± 0.00 a	13.53 ± 0.25 a	13.55 ± 0.00 a	13.70 ± 0.00 a	13.45 ± 0.14 a	13.70 ± 0.00 a
**Volatile acidity (g/L)**	0.33 ± 0.01 ab	0.34 ± 0.01 ab	0.35 ± 0.04 ab	0.32 ± 0.01 a	0.33 ± 0.02 ab	0.37 ± 0.01 b	0.43 ± 0.04 a	0.41 ± 0.00 a	0.41 ± 0.01 a	0.44 ± 0.01 a	0.42 ± 0.01 a	0.45 ± 0.01 a
**F-SO_2 (mg/L)**	4 ± 0 ab	9 ± 3 b	6 ± 1 ab	5 ± 2 ab	3 ± 0 a	7 ± 5 ab	11 ± 2 ab	18 ± 2 b	7 ± 3 a	16 ± 5 b	6 ± 0.1a	14 ± 2 b
**T-SO_2 (mg/L)**	58 ± 3 ab	67 ± 4 b	50 ± 8a	52 ± 6 ab	45 ± 11 a	66 ± 3 b	48 ± 1 a	64 ± 0 c	49 ± 10 a	57 ± 0 a	48 ± 11 a	64 ± 9 a

Values with a different letter in the same row indicate statistically significant differences ($p<0.05$).

Table S2. Concentration of volatile compounds of the different *Verdejo* and *Sauvignon blanc* wines studied.

	Verdejo												*Sauvignon blanc*											
	**W 3**		**DEOX 3**		**W 6**		**DEOX 6**		**W 8.4**		**DEOX 8.4**		**W 3**		**DEOX 3**		**W 6**		**DEOX 6**		**W 8.4**		**DEOX 8.4**	
ALCOHOLS (µg /L)																								
Isobutanol	2123	b	2123	b	1936	ab	2032	b	2031	b	1757	a	1617	a	1539	a	1484	a	1468	a	1552	a	1617	a
1-Butanol	142	a	130	a	126	a	130	a	129	a	120	a	217	a	197	a	195	a	194	a	198	a	205	a
Isoamyl aLcohols	46340	b	43472	b	42455	ab	43176	b	42559	ab	38418	a	43518	a	38867	a	40184	a	37476	a	39895	a	39945	a
3-methyl-1-pentanol	61.7	b	61.9	b	59.8	ab	59.8	ab	58.7	ab	57.0	a	48.8	a	51.4	a	51.1	a	51.8	a	52.0	a	52.9	a
Benzyl alcohol	254	bc	247	abc	242	abc	258	c	238	ab	233	a	273	a	256	a	258	a	251	a	262	a	264	a
Phenylethyl alcohol	80172	c	78499	bc	76015	b	76747	bc	76785	bc	70363	a	69084	a	64481	a	67584	a	62304	a	67144	a	66030	a
1hexanol	3332	c	3252	bc	3134	ab	3195	abc	3144	ab	3058	a	3193	a	3064	a	3066	a	2990	a	3131	a	3112	a
Cis-3-hexen-1-ol	199	b	189	ab	187	a	190	ab	186	a	180	a	205	a	196	a	197	a	192	a	201	a	199	a
Metionol	262	bc	254	abc	247	ab	268	c	246	ab	237	a	207	b	191	ab	192	ab	182	a	194	ab	197	ab
Tyrosol	1620	ab	1748	cd	1590	a	1818	b	1671	abc	1686	bc	1675	a	1721	a	1632	a	1753	a	1706	a	1864	a
**Total alcohols**	**134506**	**b**	**129976**	**b**	**125992**	**b**	**127874**	**b**	**127048**	**b**	**116109**	**a**	**120038**	**a**	**110563**	**a**	**114843**	**a**	**106862**	**a**	**114335**	**a**	**113486**	**a**
ALCOHOL ACETATES (µg/L)																								
Isoamyl acetate	1875	d	1745	bc	1775	c	1676	ab	1768	bc	1618	a	1642	a	1555	a	1591	a	1551	a	1587	a	1582	a
Hexyl acetate	158	b	144	a	147	a	142	a	145	a	141	a	148	a	144	a	144	a	146	a	148	a	146	a
Phenylethyl acetate	999	bc	1014	c	989	b	956	a	986	b	987	b	764	a	755	a	791	a	754	a	796	a	764	a
**Total alcohol acetates**	3033	**c**	**2902**	**b**	**2911**	**b**	**2774**	**a**	**2900**	**b**	**2747**	**a**	**2554**	**a**	**2454**	**a**	**2526**	**a**	**2451**	**a**	**2531**	**a**	**2492**	**a**
ETHYL ESTERS (µg/L)																								
Ethyl hexanoate	695	c	632	ab	646	b	616	ab	640	ab	612	a	508	a	493	a	491	a	496	a	507	a	495	a
Ethyl octanoate	1081	b	877	a	931	a	877	a	944	a	934	a	631	a	601	a	584	a	587	a	604	a	575	a
Ethyl decanoate	224	c	150	a	184	b	141	a	193	b	198	b	91	a	93	a	84	a	87	a	92	a	84	a
Diethyl succinate	877	bc	893	c	864	ab	845	a	880	bc	868	ab	497	a	513	a	538	a	512	a	539	a	519	a
Ethyl lactate	757	b	731	ab	708	ab	756	b	715	ab	667	a	828	a	770	a	768	a	746	a	771	a	790	a
Monoethyl succinate	1363	bc	1260	ab	1321	abc	1403	c	1290	ab	1229	a	1445	a	1310	a	1327	a	1311	a	1362	a	1394	a
**Total ethyl esters**	**4996**	**b**	**4544**	**a**	**4654**	**a**	**4639**	**a**	**4662**	**a**	**4507**	**a**	**4000**	**a**	**3781**	**a**	**3792**	**a**	**3740**	**a**	**3876**	**a**	**3858**	**a**
ACIDS (µg/L)																								

Propanoic acid	4.88	c	4.57	b	4.22	a	4.81	c	4.22	a	4.21	a	5.40	a	5.53	a	5.25	a	5.58	a	5.35	a	5.85	a
Butyric acid	52.3	bc	52.2	bc	49.1	ab	53.9	c	49.6	abc	47.6	a	53.4	a	49.7	a	48.8	a	49.1	a	50.0	a	51.7	a
Isobutyric acid	27.1	ab	28.4	b	25.2	a	28.1	b	25.7	a	25.6	a	34.4	a	33.1	a	33.1	a	33.3	a	33.8	a	34.8	a
Hexanoic acid	1658	c	1611	bc	1579	b	1600	bc	1579	b	1499	a	1350	ab	1261	a	1313	a	1238	a	1304	a	1291	a
Octanoic acid	3717	c	3730	c	3622	b	3458	a	3616	b	3454	a	2561	ab	2540	a	2739	b	2552	ab	2696	ab	2604	ab
Decanoic acid	770	cd	777	d	760	c	719	a	742	b	736	b	513	a	521	a	562	a	532	a	555	a	521	a
Isovaleric acid	70.2	a	67.1	a	65.0	a	69.6	a	66.6	a	63.3	a	65.0	a	58.4	a	59.1	a	57.6	a	60.3	a	60.7	a
**Total acids**	**6299**	**d**	6271	d	**6105**	c	**5933**	**b**	**6083**	**c**	**5830**	**a**	**4583**	**a**	**4469**	**a**	**4760**	**a**	**4468**	**a**	**4705**	**a**	**4569**	a
ALDEHYDES (μg/L)																								
Benzaldehyde	34.2	ab	34.7	ab	34.4	ab	33.4	a	35.2	b	33.7	ab	0.79	a	0.87	a	0.77	a	0.84	a	0.81	a	1.08	b
VANILLIC DERIVATIVES (μg/L)																								
Methyl vanillate	15.9	a	16.3	a	16.2	a	16.0	a	15.9	a	16.1	a	9.1	a	9.3	a	9.7	a	9.5	a	9.5	a	9.8	a
Vanillyl acetone	43.8	a	43.8	a	43.5	a	43.2	a	43.3	a	43.9	a	24.1	a	24.8	a	26.2	a	25.0	a	25.4	a	25.4	a
Acetovanillone	85.9	a	86.4	a	85.5	a	86.8	a	84.6	a	85.2	a	37.1	a	37.5	a	37.7	a	36.4	a	37.3	a	38.2	a
**Total vanillic derivatives**	**146**	**a**	147	a	**145**	a	**146**	**a**	**144**	**a**	**145**	**a**	**70.3**	**a**	**71.6**	**a**	**73.5**	**a**	**70.9**	**a**	**72.3**	**a**	**73.4**	a
LACTONES (μg/L)																								
γ-butyrolactone	65.7	b	65.5	ab	63.5	ab	68.1	b	63.6	ab	59.7	a	89.6	a	85.3	a	85.0	a	82.8	a	86.8	a	87.4	a
NORISOPRENOIDS (μg/L)																								
β-damascenone	10.9	b	10.4	a	10.0	a	10.3	a	10.2	a	10.5	ab	7.49	a	7.79	a	8.05	a	7.77	a	7.92	a	7.79	a
PHENOLS (μg/L)																								
Eugenol	5.92	a	5.89	a	5.95	a	5.82	a	5.92	a	5.99	a	3.70	a	3.83	ab	4.12	b	3.92	ab	4.14	b	3.92	ab
4-vinylguaiacol	283	ab	291	a	283	ab	281	a	284	ab	284	ab	31.9	a	33.4	a	34.8	a	34.0	a	34.9	a	34.1	a
**Total phenols**	**289**	**ab**	297	b	**289**	ab	**287**	**a**	**290**	**ab**	**290**	**ab**	**35.6**	**a**	**37.2**	**ab**	**38.9**	**ab**	**37.9**	**ab**	**39.0**	**b**	**38.0**	ab
TERPENES (μg/L)																								
Trans-geraniol	4.60	a	4.85	a	4.64	a	4.62	a	4.78	a	4.46	a	5.67	a	6.19	ab	6.29	ab	6.31	ab	6.58	b	6.23	ab

Values with a different letter in the same row indicate statistically significant differences (p<0.05).

Table S3. Odor threshold and odor active values (OAVs) of each volatile compound of the different *Verdejo* and *Sauvignon blanc* wines studied.

		Verdejo						*Sauvignon blanc*					
		**W 3**	**DEOX 3**	**W 6**	**DEOX 6**	**W 8.4**	**DEOX 8.4**	**W 3**	**DEOX 3**	**W 6**	**DEOX 6**	**W 8.4**	**DEOX 8.4**
ALCOHOLS (mg/L)	Threshold (µg/L)	OAV	OAV	OAV	OAV	OAV	OAV	OAV	OAV	OAV	OAV	OAV	OAV
Isobutanol	40000	0.05	0.05	0.05	0.05	0.05	0.04	0.04	0.04	0.04	0.04	0.04	0.04
1-Butanol	150000	0.00	0.00	0.00	0.00	0.00	0.00	0.00	0.00	0.00	0.00	0.00	0.00
Isoamyl aLcohols	60000	0.77	0.72	0.71	0.72	0.71	0.64	0.73	0.65	0.67	0.62	0.66	0.67
3-methyl-1-pentanol	n.f.												
Benzyl alcohol	200000	0.00	0.00	0.00	0.00	0.00	0.00	0.00	0.00	0.00	0.00	0.00	0.00
Phenylethyl alcohol	14000	**5.73**	**5.61**	**5.43**	**5.48**	**5.48**	**5.03**	**4.93**	**4.61**	**4.83**	**4.45**	**4.80**	**4.72**
1hexanol	8000	0.42	0.41	0.39	0.40	0.39	0.38	0.40	0.38	0.38	0.37	0.39	0.39
Cis-3-hexen-1-ol	400	0.50	0.47	0.47	0.48	0.47	0.45	0.51	0.49	0.49	0.48	0.50	0.50
Metionol	1	**262**	**254**	**247**	**268**	**246**	**237**	**207**	**191**	**192**	**182**	**194**	**197**
Tyrosol	100000	0.02	0.02	0.02	0.02	0.02	0.02	0.02	0.02	0.02	0.02	0.02	0.02
ALCOHOL ACETATES (µg/L)													
Isoamyl acetate	30	**62.50**	**58.17**	**59.17**	**55.87**	**58.93**	**53.93**	**54.73**	**51.83**	**53.03**	**51.70**	**52.90**	**52.73**
Hexyl acetate	670	0.24	0.21	0.22	0.21	0.22	0.21	0.22	0.21	0.21	0.22	0.22	0.22
Phenylethyl acetate	250	**4.00**	**4.06**	**3.96**	**3.82**	**3.94**	**3.95**	**3.06**	**3.02**	**3.16**	**3.02**	**3.18**	**3.06**
ETHYL ESTERS (µg/L)													
Ethyl hexanoate	5	**139**	**126**	**129**	**123**	**128**	**122**	**102**	**99**	**98**	**99**	**101**	**99**
Ethyl octanoate	2	**541**	**439**	**466**	**439**	**472**	**467**	**316**	**301**	**292**	**294**	**302**	**288**
Ethyl decanoate	200	1.12	0.75	0.92	0.71	0.97	0.99	0.46	0.47	0.42	0.44	0.46	0.42
Diethyl succinate	1200	0.73	0.74	0.72	0.70	0.73	0.72	0.41	0.43	0.45	0.43	0.45	0.43
Ethyl lactate	150000	0.01	0.00	0.00	0.01	0.00	0.00	0.01	0.01	0.01	0.00	0.01	0.01
Monoethyl succinate	n.f.												
ACIDS (µg/L)													
Propanoic acid	8200	0.00	0.00	0.00	0.00	0.00	0.00	0.00	0.00	0.00	0.00	0.00	0.00
Butyric acid	173	0.30	0.30	0.28	0.31	0.29	0.28	0.31	0.29	0.28	0.28	0.29	0.30
Isobutyric acid	200000	0.00	0.00	0.00	0.00	0.00	0.00	0.00	0.00	0.00	0.00	0.00	0.00
Hexanoic acid	3000	0.55	0.54	0.53	0.53	0.53	0.50	0.45	0.42	0.44	0.41	0.43	0.43
Octanoic acid	500	**7.43**	**7.46**	**7.24**	**6.92**	**7.23**	**6.91**	**5.12**	**5.08**	**5.48**	**5.10**	**5.39**	**5.21**
Decanoic acid	1000	0.77	0.78	0.76	0.72	0.74	0.74	0.51	0.52	0.56	0.53	0.56	0.52
Isovaleric acid	3000	0.02	0.02	0.02	0.02	0.02	0.02	0.02	0.02	0.02	0.02	0.02	0.02

ALDEHYDES (μg/L)													
Benzaldehyde	2000	0.02	0.02	0.02	0.02	0.02	0.02	0.00	0.00	0.00	0.00	0.00	0.00
VANILLIC DERIVATIVES													
Methyl vanillate	3000	0.01	0.01	0.01	0.01	0.01	0.01	0.00	0.00	0.00	0.00	0.00	0.00
Vanillyl acetone	n.f.												
Acetovanillone	1000	0.09	0.09	0.09	0.09	0.08	0.09	0.04	0.04	0.04	0.04	0.04	0.04
LACTONES (μg/L)													
γ-butyrolactone	n.f.												
NORISOPRENOIDS (μg/L)													
β-damascenone	0.05	**218**	**208**	**200**	**206**	**204**	**210**	**150**	**1556**	**161**	**155**	**158**	**156**
PHENOLS (μg/L)													
Eugenol	5	**1.18**	**1.18**	**1.19**	**1.16**	**1.18**	**1.20**	0.74	0.77	0.82	0.78	0.83	0.78
4-vinylguaiacol	40	**7.08**	**7.28**	**7.08**	**7.03**	**7.10**	**7.10**	0.80	0.84	0.87	0.85	0.87	0.85
TERPENES (μg/L)													
Trans-geraniol	20	0.23		0.23	0.23	0.24	0.22	0.28	0.31	0.31	0.32	0.33	0.31

Table S4. Increasing or decreasing concentration (μg/L) of volatile compounds during the aging in bottle of the different Verdejo and Sauvignon blanc wines studied.

	Verdejo						*Sauvignon blanc*					
	**W 3**	**DEOX 3**	**W 6**	**DEOX 6**	**W 8.4**	**DEOX 8.4**	**W 3**	**DEOX 3**	**W 6**	**DEOX 6**	**W 8.4**	**DEOX 8.4**
ALCOHOLS												
Isobutanol	208 a	243 a	276 a	199 a	168 a	363 a	602 a	676 a	630 a	515 a	598 a	309 a
1-Butanol	4.71 a	14.57 a	5.87 a	0.04 a	-3.37 a	1.93 a	67.9 a	74.7 a	71.2 a	38.4 a	55.8 a	63.8 a
Isoamyl aLcohols	12057 a	12312 a	12125 a	10915 a	12853 a	12942 a	23273 ab	21536 ab	26162 b	15204 a	19559 ab	14115 a
3-methyl-1-pentanol	34.9 a	32.6 a	45.2 a	46.2 a	45.0 a	46.6 a	49.3 c	43.7 bc	22.3 a	34.7 ab	44.6 bc	36.7 abc
Benzyl alcohol	131 a	125 a	127 a	110 a	144 a	92 a	166 b	195 b	169 b	112 a	166 b	115 a
Phenylethyl alcohol	56169 a	52348 a	54628 a	53260 a	54880 a	49987 a	54872 b	51884 b	52444 b	38074 a	47224 ab	39025 a
1hexanol	1124 a	947 a	1117 a	1014 a	1040 a	871 a	1533 a	1522 a	1656 a	1271 a	1485 a	1136 a
Cis-3-hexen-1-ol	-36.3 a	-39.0 a	-46.2 a	-55.3 a	-31.8 a	-55.3 a	6.30 b	6.24 b	2.03 ab	-21.0 a	7.11 b	-15.6 ab
Metionol	38.6 a	57.9 a	51.2 a	43.0 a	50.1 a	46.3 a	79 cd	84 cd	105 d	47 ab	69 bc	33 a
Tyrosol	931 a	969 a	910 a	773 a	905 a	667 a	1286 b	1337 b	1357 b	563 a	1136 b	701 a
**Total alcohols (mg/L)**	**70.7 a**	**67.0 a**	**69.2 a**	**66.3 a**	**70.1 a**	**65.0 a**	**81.9 b**	**77.4 ab**	**82.6 b**	**55.8 a**	**70.3 ab**	**55.5 a**
ALCOHOL ACETATES												
Isoamyl acetate	-693 a	-614 a	-663 a	-584 ab	-685 ab	-501 b	-673 a	-597 a	-577 a	-606 a	-575 a	-626 a
Hexyl acetate	-89.3 a	-78.8 ab	-82.2 ab	-78.0 ab	-85.2 ab	-77.6 b	-83.4 a	-76.9 a	-77.2 a	-81.2 a	-77.2 a	-80.0 a
Phenylethyl acetate	-398 a	-412 a	-387 a	-342 b	-395 a	-409 a	-275 b	-288 ab	-335 a	-298 ab	-317 ab	-325 ab
**Total alcohol acetates**	**-1181 a**	**-1104 abc**	**-1131 abc**	**-1004 bc**	**-1164 ab**	**-988 c**	**-1032 a**	**-962 a**	**-989 a**	**-985 a**	**-969 a**	**-1031 a**
ETHYL ESTERS												
Ethyl hexanoate	-79.4 a	-23.6 ab	-59.9 ab	-20.2 ab	-65.6 ab	-8.64 b	-47.5 a	-38.1 a	-1.88 a	-65.0 a	-18.5 a	-60.6 a
Ethyl octanoate	-259 a	-105 c	-166 bc	-120 c	-217 ab	-159 bc	-151 a	-120 a	-84 a	-146 a	-95 a	-128 a
Ethyl decanoate	-115 a	-56.4 d	-74.2 c	-30.1 e	-101 b	-83.2 c	-44.7 a	-41.6 a	-39.8 a	-39.2 a	-37.4 a	-39.7 a
Diethyl succinate	1590 a	1465 a	1487 a	1483 a	1582 a	1403 a	2312 c	2084 b	2202 bc	1887 a	2109 b	1790 a
Ethyl lactate	880 a	929 a	926 a	890 a	962 a	808 a	1291 b	1269 b	1321 b	980 a	1208 ab	993 a
Monoethyl succinate	2554 b	2562 b	2350 ab	2149 ab	2509 b	2034 a	3610 c	3131 b	3558 bc	2437 a	3197 bc	2493 a
**Total ethyl esters**	**4570 a**	**4771 a**	**4463 a**	**4352 a**	**4670 a**	**3995 a**	**6969 c**	**6285 b**	**6955 c**	**5053 a**	**6363 b**	**5048 a**
ACIDS												
Propanoic acid	1.28 a	1.79 ab	1.76 ab	1.28 a	2.25 b	1.32 a	3.88 b	3.50 b	3.88 b	1.55 a	3.08 b	1.92 a
Butyric acid	31 a	29 a	31 a	24 a	34 a	25 a	38.2 b	39.3 b	42.5 b	23.6 ab	37.5 b	21.1 a
Isobutyric acid	-6.85 a	-8.14 a	-5.73 a	-8.97 a	-5.76 a	-7.60 a	-13.0 ab	-13.0 ab	-12.4 b	-16.3 ab	-14.4 ab	-17.1 a
Hexanoic acid	433 a	402 a	418 a	416 a	465 a	370 a	527 c	493 c	504 c	315 ab	433 bc	285 a
Octanoic acid	-447 a	-487 a	-476 a	-205 b	-482 a	-327 ab	79.3 d	-77.1 c	-160 bc	-224 ab	-183 abc	-302 a
Decanoic acid	-356 ab	-360 a	-364 a	-315 b	-352 ab	-329 ab	-197 b	-194 b	-272 a	-229 ab	-211 b	-229 ab
Isovaleric acid	31.6 a	32.6 a	33.6 a	27.8 a	36.0 a	26.8 a	33.8 b	37.5 b	40.2 b	19.4 a	34.0 b	21.3 a

**Total acids**	**-314**	**a**	**-390**	**a**	**-361**	**a**	**-59**	**a**	**-303**	**a**	**-241**	**a**	**472**	**d**	**290**	**cd**	**147**	**c**	**-110**	**ab**	**99**	**bc**	**-219**	**a**
ALDEHYDES																								
Benzaldehyde	60.1	bc	38.3	a	48.8	ab	44.0	a	61.2	c	37.0	a	1.4	b	0.8	a	1.5	b	0.7	a	1.2	b	0.5	a
VANILLIC DERIVATIVES																								
Methyl vanillate	-2.25	a	-0.92	ab	-1.46	ab	-1.75	ab	-0.18	b	-1.69	ab	0.96	c	-0.09	bc	-0.18	bc	-0.78	ab	1.12	c	-1.90	a
Vanillyl acetone	0.10	b	-0.79	ab	-0.41	ab	0.05	b	-0.79	ab	-2.43	a	3.68	c	3.05	bc	2.09	abc	-0.69	a	3.70	c	-0.11	ab
Acetovanillone	11.9	a	13.1	a	7.6	a	9.4	a	11.9	a	4.3	a	8.73	cd	6.31	bc	8.10	cd	2.90	ab	10.4	d	1.81	a
**Total vanillic derivatives**	**9.8**	**ab**	**11.4**	**b**	**5.7**	**ab**	**7.7**	**ab**	**10.9**	**ab**	**0.21**	**a**	**13.4**	**b**	**9.28**	**b**	**10.0**	**bc**	**1.44**	**a**	**15.2**	**b**	**-0.20**	**a**
LACTONES																								
γ-butyrolactone	45.0	b	34.8	ab	40.9	ab	31.5	ab	42.7	ab	29.9	a	52.1	b	58.9	b	53.8	b	37.4	a	55.8	b	36.5	a
NORISOPRENOIDS																								
β-damascenone	-0.24	ab	-0.67	ab	0.16	bc	0.19	bc	1.67	c	-1.56	a	9.56	c	7.85	b	10.2	c	5.50	a	9.81	c	4.96	a
PHENOLS																								
Eugenol	-0.28	a	-0.13	a	-0.31	a	0.46	a	-0.43	a	-0.24	a	0.53	b	0.47	b	-0.44	a	-0.19	ab	-0.04	ab	-0.18	ab
4-vinylguaiacol	-131	b	-168	b	-141	bc	-144	a	-132	b	-165	a	4.71	c	5.00	c	-1.73	ab	0.07	b	-1.16	ab	-4.86	a
**Total phenols**	**-131**	**b**	**-168**	**a**	**-141**	**b**	**-144**	**b**	**-133**	**b**	**-165**	**a**	**5.24**	**c**	**5.46**	**c**	**-2.17**	**ab**	**-0.12**	**b**	**-1.20**	**ab**	**-5.04**	**a**
TERPENES																								
Trans-geraniol	nd		nd		nd		nd		nd		nd		nd		nd		nd		nd		nd		nd	

Values with a different letter in the same row indicate statistically significant differences (p<0.05).

The increase or decrease in the concentration of each compound has been calculated by taking the difference between the value after 6 months in the bottle minus the value after sparging.
